# A redox-dependent switch governing sensory axon degeneration and regeneration

**DOI:** 10.1038/s41598-025-23035-6

**Published:** 2025-11-11

**Authors:** Chia-Jung Hsieh, Lauryn M. Lee, Sandra Rieger

**Affiliations:** 1 Department of Biology, University of Miami, Coral Gables, FL 33146 USA; 2 Sylvester Comprehensive Cancer Center, Miller School of Medicine, University of Miami, Miami, FL 33136 USA

**Keywords:** Peroxynitrite, Axon degeneration, Axon regeneration, Chemotherapy-induced peripheral neuropathy, Paclitaxel, NADPH, Nitrosylation, Nitration, Sensory neuron, Diseases of the nervous system, Peripheral nervous system, Regeneration and repair in the nervous system, Somatosensory system

## Abstract

Zebrafish (*Danio rerio*) fin amputation is a widely used model for studying sensory axon degeneration and regeneration. After injury, sensory neuron terminals rapidly degenerate before regenerating. While reactive oxygen species (ROS), particularly hydrogen peroxide (H₂O₂), are known to promote axon regeneration through epidermal mechanisms, their role in degeneration remains unclear. Here, we identify mitochondrial superoxide and reactive nitrogen species (RNS), specifically peroxynitrite, as key drivers of axon fragmentation. Using AlphaFold and DeepNitro predictive modeling, we identified conserved nitration and nitrosylation sites in NMNAT rather than SARM1, two key drivers of a known axon destruction pathway, suggesting the possibility of a redox-dependent regulatory mechanism. We further explored the role of NADPH in axon degeneration since *cyba* mutants that do not utilize NADPH to generate ROS display delayed sensory axon degeneration. Pharmacological NADPH treatment significantly reduced amputation-induced sensory axon degeneration while enhancing regeneration. NADPH co-administration also mitigated paclitaxel-induced axon loss and improved the tactile response in a model of chemotherapy-induced peripheral neuropathy. These findings reveal a complex interplay between ROS and RNS in axon degeneration and regeneration, positioning NADPH as a promising therapeutic candidate for oxidative stress-related neurodegeneration.

**Supplementary Information:**

The online version contains supplementary material available at 10.1038/s41598-025-23035-6.

## Introduction

Skin injury triggers a cascade of events that facilitate wound closure and remodeling, including sensory axon degeneration and regeneration^1^, apoptotic signaling^2^, immune cell recruitment^3,4^, keratinocyte-mediated re-epithelialization^5,6^, and extracellular matrix deposition^7^. While the cellular and molecular mechanisms underlying many aspects of wound repair are well characterized, the regulation of early sensory axon degeneration (axon die-back) in skin wounds remains largely unexplored. The skin is innervated by peripheral axons of somatosensory neurons of either trigeminal or dorsal root ganglion (DRG) origin^8^. Most studies on trigeminal and DRG neurons focused on degeneration pertaining to severing of the nerve, either via crush injury or axotomy in which the nerve or cutaneous axon terminals are completely severed. These studies have revealed that the distal axonal segments that are separated from the soma undergo a controlled process of Wallerian degeneration (WD). First described by Augustus Waller in 1850^9^, WD involves the activation of SARM1 (Sterile alpha and TIR motif-containing protein 1) in axons due to the degradation of the enzyme, NMNAT (NAM Adenylyl Transferase)^10–15^. Under homeostatic conditions, axonal NMNAT converts nicotinamide mononucleotide (NMN) into nicotinamide adenine dinucleotide (NAD^+^) using ATP^16^. NAD^+^ binds to SARM1 attached to the outer mitochondria membrane^17,18^ to maintain an inactive state^18^. Following nerve injury, NMNAT is degraded in axons, leading to the depletion of NAD^+^ and accumulation of NMN, which increasingly binds to SARM1 while NAD^+^ is metabolized into nicotinamide and ADP-ribose^19^. Subsequently, a cascade of downstream events is activated that culminates in calcium influx and Annexin-mediated membrane exposure that stimulates axon fragmentation^20–22^. This pathway has also been suggested to play a role in the central nervous system in neurodegenerative diseases, such as amyotrophic lateral sclerosis^23^, demonstrating that axon destruction via NMNAT/SARM1 is conserved.

Studies investigating cutaneous sensory axon de- and regeneration behavior in zebrafish have shown that laser axotomy of unmyelinated Rohon-Beard (RB) sensory nerve endings induces WD^24^. This model also demonstrated that mitochondria in axotomized distal axons produce H_2_O_2_. Thus, ROS production may play a crucial role in axon degeneration. Moreover, elevated mitochondrial ROS (mitoROS) induce axon degeneration in cultured mouse DRG neurons, but not in SARM1^−/−^ knockout neurons treated with the mitochondrial ROS inducers, CCCP and rotenone^11^. Thus, ROS may play a critical role in cutaneous sensory axon degeneration. Specifically, reactive nitrogen species (RNS) that are produced downstream of ROS via nitric oxide (NO) and nitric oxide synthase (NOS) have been linked to axon degeneration in various neurodegenerative diseases. RNS can nitrate cysteine residues or nitrosylate tyrosine and tryptophan residues, leading to conformational changes in proteins that alter their functions or activity^25^. For instance, nitrotyrosine was more abundant in the brain of patients with Alzheimer’s disease due to increased peroxynitrite formation^26^, similar to patients with Parkinson’s disease^27^. RNS have been preferentially implicated in axon degenerative conditions, whereas physiological H_2_O_2_ production following injury stimulates axon regeneration^1,28,29^. How ROS/RNS differentially regulate axon de- and regeneration is largely unknown. ROS can be formed either as a byproduct in the electron transport chain during mitochondrial ATP formation, or by NADPH oxidases, which utilize NADPH to transfer an electron to molecular oxygen, leading to superoxide (O_2_^−^) production. Superoxide is either directly converted into H_2_O_2_ by the same NADPH oxidase (i.e. Duox and Nox4) or it is converted by superoxide dismutase (SOD) into other metabolites such as sulfinic and sulfonic acid, or hydrogen peroxide (H_2_O_2_) (i.e. Nox1-3, and Nox5)^30,31^. Alternatively, superoxide can react with NO that is generated by NOS to generate peroxynitrite (ONOO^−^)^25^. While ROS and RNS production intersects and relies on the same upstream mechanisms, their distinct biochemical properties likely drive opposing effects in injured axons, with ROS primarily supporting regeneration and RNS contributing to degeneration.

Using live imaging in amputated larval zebrafish, we demonstrate that neuronal mitoROS and peroxynitrite play a critical role in sensory axon degeneration with NMNAT being a potential target for RNS, in contrast to the established role of H₂O₂ in promoting axon regeneration. Furthermore, we uncovered that NADPH treatment delays axon degeneration in both injury conditions and chemotherapy-induced peripheral neuropathy, suggesting its potential as a therapeutic strategy for mitigating axonal loss.

## Results

### Cutaneous sensory axon degeneration is enhanced by increased MitoROS following skin wounding

**Fig. 1:**
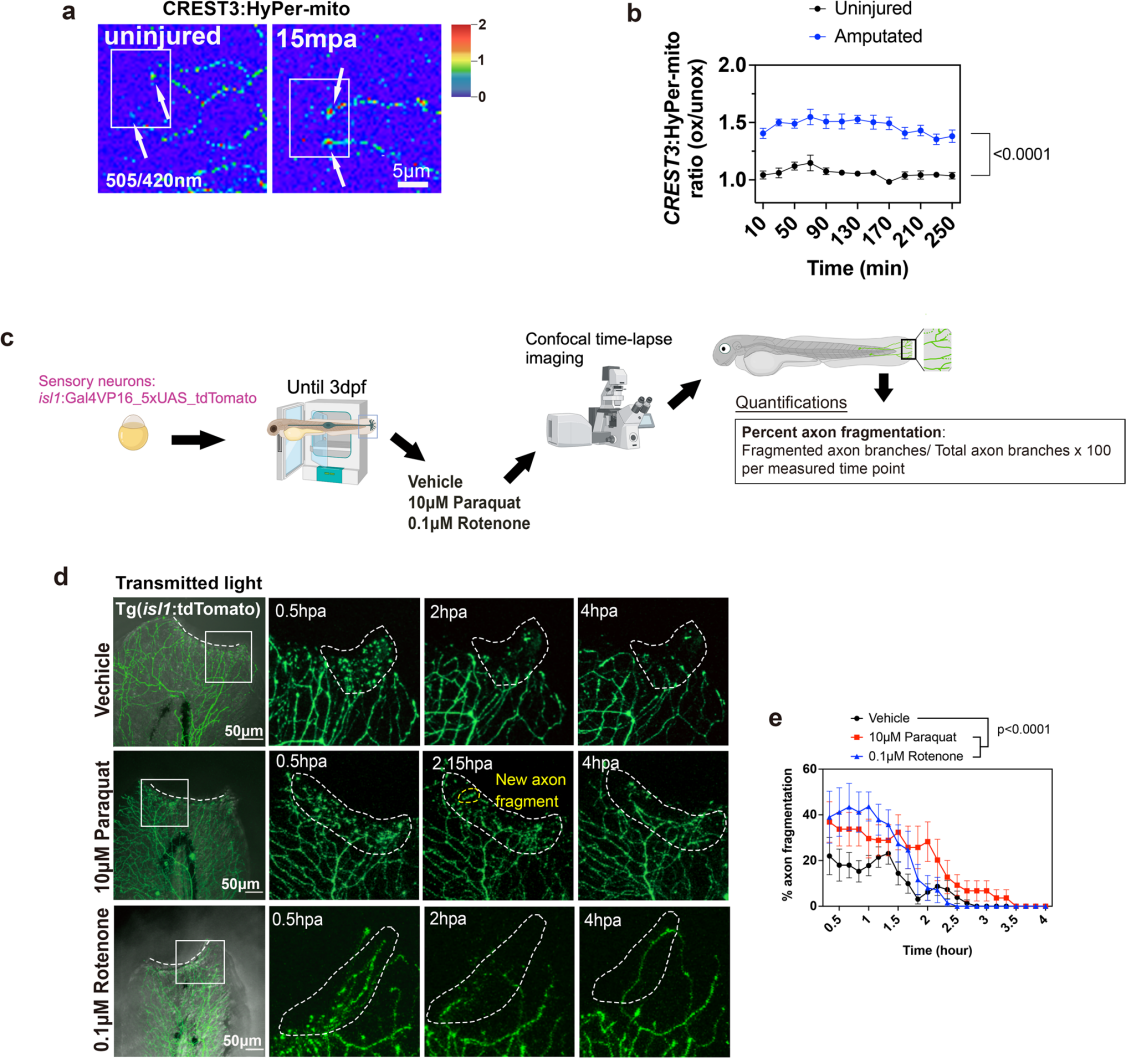
mitoROS induce cutaneous sensory axon degeneration. (**a**) CREST3:HyPer-mito expression in cutaneous sensory neurons shows increased mitochondrial HyPer oxidation in axon tips close to the amputation plane (toward left), as compared to mitochondria in uninjured axon tips (arrows). (**b**) Quantification over the course of 4 h shows increased mitochondrial HyPer oxidation following amputation compared to uninjured axons (Uninjured: *n* = 6, Amputated: *n* = 5). (**c**) Experimental procedure. (**d**) Axons were visualized in Tg(*isl1*:lexA-lexAop_tdTomato) fish to monitor axon fragmentation. Dashed lines indicate the amputation plane whereas dashed circles show areas in which axons fragmented following amputation of vehicle, 10µM paraquat, and 0.1µM rotenone. 0.5 h and 4 h post amputation (hpa) is shown. Yellow dashed circle shows new fragmentation onset at 2.15 h. (**e**) Quantification of the percentage of axon fragmentation between 0-4hpa (*n* = 9, vehicle: *n* = 10, vehicle: *n* = 10). Two-way ANOVA and Tukey’s post-test was used for all comparisons. Significance: *p* < 0.05.

Cutaneous sensory axons follow a stereotypical response to injury. First, axon terminals die back (degenerate), which is followed by axon regeneration and reinnervation of the wound^1,32,33^. ROS formation has been shown to be a key event in mitochondria of axotomized sensory axons^24^, but its relevance has not been explored. We asked two questions: (1) Are mitoROS formed in axons following amputation of the caudal fin, which was not tested, and (2) do mitoROS play a role in axon degeneration. To examine the first question, we expressed the transgene, HyPer-mito detecting H_2_O_2_ formation, under the CREST3 promoter in RB sensory neurons for ratiometric quantification of mitoROS in the absence and presence of fin amputation. Caudal fin amputation at 3 days post fertilization (dpf) damages several major cell types: epidermal keratinocytes, sensory axons, and mesenchymal cells^34^. Microinjection of CREST3:HyPer-mito into newly fertilized wildtype embryos resulted in mosaic expression in differentiated sensory neurons at 3dpf, which enabled us to track only a few axonal mitochondria among the extensive axonal network present in the skin at that age^1^. Time-lapse imaging for 4 h post amputation (hpa) showed a significant increase in mitoROS/H_2_O_2_ in amputated axon tips, but not in uninjured control fish (Fig. 1a, b). Interestingly, mitoROS/H_2_O_2_ levels were sustained over several hours particularly in regenerating axons, indicating that these might be primarily responsible to drive axon regeneration.

To further determine whether mitochondrial ROS/H_2_O_2_ play a role in sensory axon degeneration, we treated 3-day old Tg(*isl1*:lexA-lexAop_tdTomato) (courtesy of Alvaro Sagasti, UCLA) zebrafish in which all cutaneous sensory axons were fluorescently labeled with red fluorescent tdTomato, with the mitoROS inducers, paraquat and rotenone, starting 30 min prior to amputation and ending at 4hpa (Fig. 1c). Paraquat induces mitoROS production mildly through redox cycling and membrane depolarization, while rotenone robustly increases mitoROS by directly inhibiting mitochondrial complex I^35^. We tested both compounds to control for mitoROS induction via two distinct pathways. First, we determined the maximum tolerated dose (MTD) for each compound in 3dpf amputated larval fish using serial dilutions. This showed that 100% of the fish survived in 10µM paraquat and 0.1µM rotenone. Examining axons under these conditions showed that significantly more axons fragmented in the rotenone and paraquat treatment compared with vehicle controls. In addition, paraquat prolonged the degeneration phase until 4hpa (Fig. 1d, e). One possibility could be differences in the ROS generation mechanisms, whereby paraquat promotes gradual superoxide accumulation, while rotenone causes rapid mitochondrial dysfunction and ATP collapse through complex I inhibition^36–38^. Together, these data suggest that mitoROS are present in sensory axons following amputation injury and enhance cutaneous sensory axon fragmentation.

### Mitochondrial superoxide stimulates axon degeneration

**Fig. 2:**
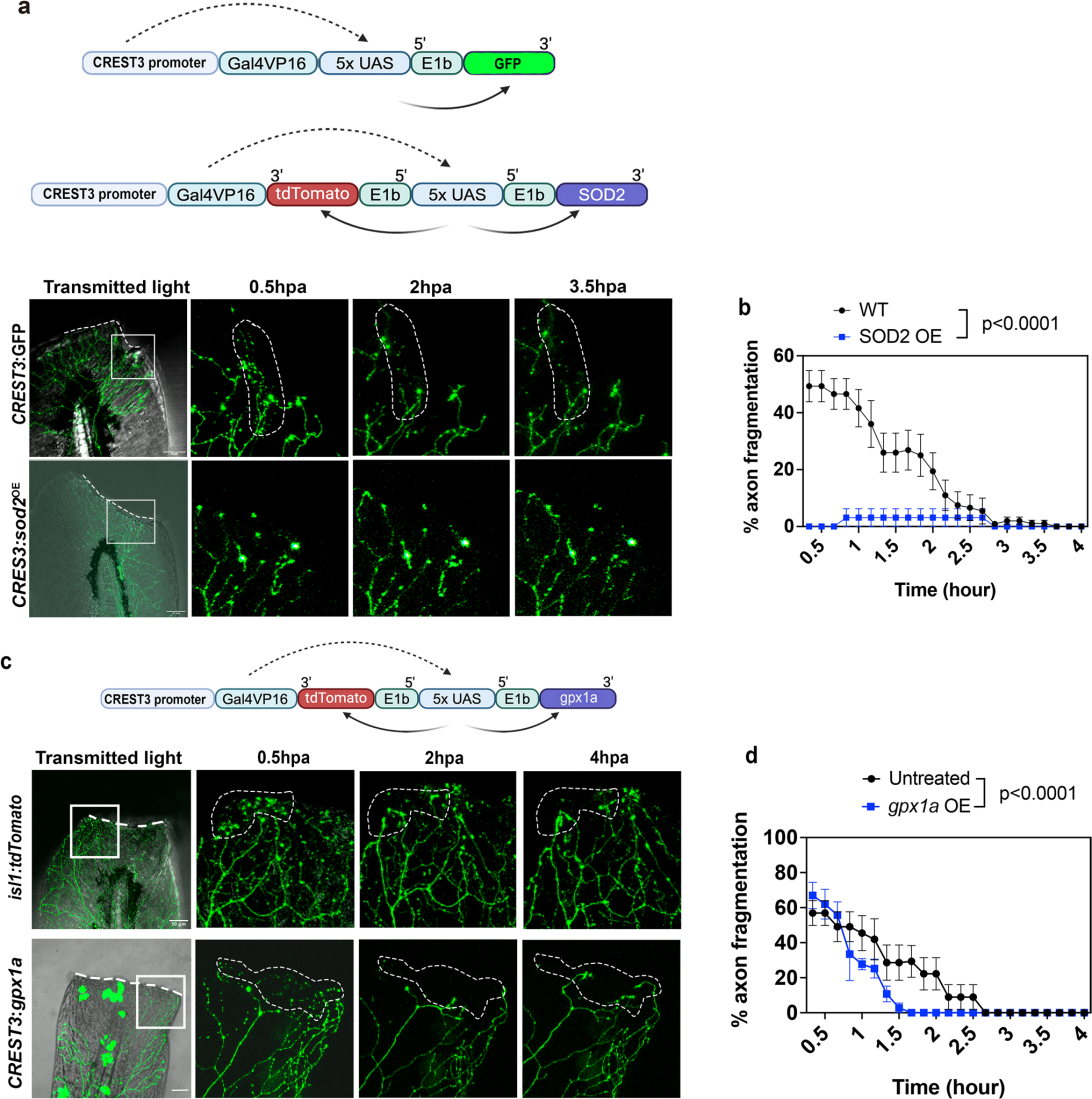
Sod2 overexpression in mitochondria of somatosensory neurons abolishes cutaneous axon degeneration. (**a**) Schematic depicting the transgene from which *sod2 and* tdTomato are separately overexpressed and using the Gal4-UAS system driven by the sensory neuron-specific *CREST3* promotor. Axon degeneration in 3dpf wildtype fish following caudal fin amputation recorded over 4 h shows degeneration is completed ~ 2.5 h post amputation (hpa). Overexpression of *sod2* in neurons prevents axon fragmentation. (**b**) S*od2* overexpression significantly reduces the number of axon branches that undergo fragmentation (WT: *n* = 11, SOD2 OE: *n* = 8). (**c**) Gpx1 overexpression (OE) does not influence to axon fragmentation. (**d**) Fragmentation takes place at similar levels initially but is completed sooner compared with wildtype fish (Untreated: *n* = 7, *gpx1a* OE: *n* = 4). Two-way ANOVA plus Tukey’s post-test. Significance: *p* < 0.05. The amputation plane is outlined by a dashed line in the first image of each treatment group in (a) and (c). Dashed circles in other images represent the area with observed changes in axons.

Given the presence of mitoROS in injured axons, and the observation of enhanced axon fragmentation in the presence of pharmacologically elevated mitoROS, we wanted to further determine whether manipulating mitoROS in sensory neurons influences axon degeneration. For this, we cloned and overexpressed zebrafish *sod2* in mitochondria of RB sensory neurons under the CREST3 promoter. This enzyme scavenges superoxide and promotes H_2_O_2_ formation^39^. We co-expressed the red fluorescent reporter, tdTomato, from the same expression cassette using a bidirectional Gal4-UAS system for tracking of Sod2-overexpressing (Sod2^OE^) axons during degeneration (Fig. 2a). Interestingly, Sod2^OE^ overexpression in axons led to significantly reduced axon fragmentation (Fig. 2a, b). Two possibilities could account for this phenotype: First, scavenging superoxide through conversion into H_2_O_2_ may be beneficial for axons, indicating that superoxide participates in amputation-induced axon degeneration. Alternatively, increased H_2_O_2_ formation may be beneficial for axons and delay or prevent axon degeneration.

While we previously showed that exogenous H_2_O_2_ addition promotes axon regeneration^1^, we and others found that high concentrations of this ROS also induce cellular damage and axon degeneration^40^. This is in line with findings that H_2_O_2_ exhibits a concentration-dependent dual role in cells. At low, submicromolar levels, it functions as a signaling molecule by modulating the activity of target enzymes such as kinases and phosphatases^41,42^. At moderate concentrations, H₂O₂ can trigger adaptive antioxidant responses^43,44^. However, when levels exceed the capacity of these defenses, antioxidant systems become overwhelmed, resulting in oxidative stress and cellular damage^43,45^. To further explore the effects of H_2_O_2_ on axon degeneration, we scavenged this ROS through overexpression of glutathione peroxidase 1a (*gpx1a*), which is part of the antioxidant defense system besides catalase and converts H_2_O_2_ into water^46,47^. This enzyme has been shown to be located in mitochondria and the cytoplasm^46^ and thus seemed to be most relevant to our system. To further ensure its expression in somatosensory neurons and to monitor its expression, we used a fluorescent variant expressed under the CREST3 promoter whereby the tdTomato was separately expressed from a bidirectional Gal4-UAS system^40^. Time-lapse imaging and quantification of axon degeneration showed that *gpx1a* overexpression slightly shortened the time until axon fragments were cleared compared with amputated control animals. Although the presence of Gpx1a did not prevent fragmentation, it accelerated the fragmentation and clearance period (Fig. 2c, d). This finding suggests that neuronal H₂O₂ contributes to the rate of fragmentation and clearance.

### Peroxynitrite drives axon degeneration without affecting axon regeneration

**Fig. 3:**
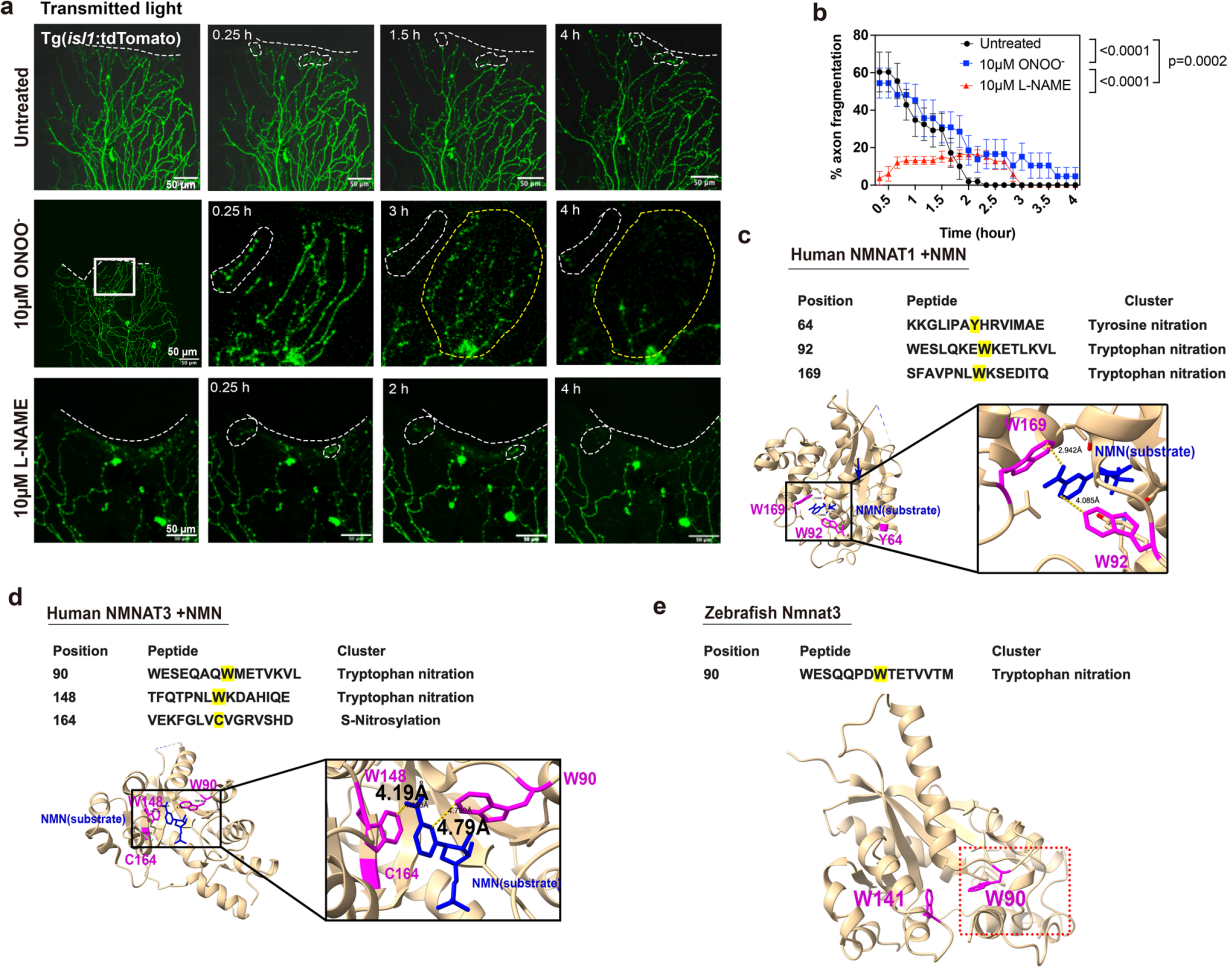
Peroxynitrite treatment prolongs axon degeneration. (**a**) Time-lapse images show axon fragmentation in Tg(*isl1*:tdTomato) fish following amputation (circular white dashed lines). Dashed line in first image of each treatment group outlines the amputation plane. The yellow dotted area displays regions of new axon fragmentation over time for ONOO^−^. (**b**) Percentage of axons that fragment over the course of 4 h following amputation (untreated: *n* = 13, 10µM ONOO-: *n* = 7, 10µM L-NAME: *n* = 6). (**c**) Human NMNAT1 crystal structure with NMN (labeled in blue) (PDB 1GZU) shows possible residues to be oxidized (tyrosine (Y64), tryptophan (W92 and 169) in magenta. Black boxes shown the enlarged NMN substrate binding region. (**d**) Human NMNAT3 crystal structure with NMN (labeled in blue) (PDB: 1NUP) shows possible residues to be oxidized (W90 and 148, and C164) in magenta. Black boxes are enlarged regions. Yellow lines indicate the distance between the substrate and the residues predicted to be nitrated. (**e**) Zebrafish Nmnat3 model based on the human crystal structure using AlphaFold shows W90 as the predicted nitration site when queried in DeepNitro (red boxed region). W141 is not predicted to be nitrated but is shown as a reference.

Superoxide is highly reactive and may react with nitric oxide to form the more stable RNS, peroxynitrite^48^. Since RNS have been associated with various neurodegenerative diseases^27,49–52^, we explored whether RNS/peroxynitrite also play a role in injury-dependent axon degeneration. Tg(*isl1*:tdTomato) zebrafish were treated at 3dpf with 10µM peroxynitrite starting 30 min prior to amputation, followed by 4 h time-lapse imaging. Peroxynitrite treatment did not significantly alter the percentage of axons that fragmented until ~ 2hpa, compared with untreated controls (Fig. 3a, b). Nevertheless, fragmentation was significantly increased thereafter, suggesting that while peroxynitrite does not further enhance fragmentation of already fragmenting axon branches (up to ~ 2-2.5hpa), it subsequently promoted the new onset of axon fragmentation. To further confirm the role of RNS in axon fragmentation, we treated fish with the non-selective NOS inhibitor, L-NAME. NOS uses L-arginine, oxygen, and Nicotinamide Adenine Dinucleotide Phosphate (NADPH) to generate nitric oxide (the smallest known signaling molecule), which then reacts with superoxide to produce peroxynitrite^53^. Zebrafish at 3dpf were treated with 10µM L-NAME starting 3 h prior to amputation until 4hpa. Imaging and quantification of amputated fins harboring tdTomato^+^ cutaneous sensory axons showed that axon fragmentation was significantly prevented (Fig. 3a, b). Together, these data indicate that superoxide/RNS are the primary driver of axon degeneration in zebrafish wounds. Since H_2_O_2_ promotes axon regeneration^1^, we wanted to examine whether also RNS play a role in regeneration. Zebrafish at 3dpf were treated with 10µM L-NAME starting 2 h prior to 12 h time-lapse imaging to monitor axon regeneration. Quantification of regrowing axon branches showed that both control and L-NAME treated zebrafish regenerated similarly (Untreated: 56.56±5.64 μm vs. 10µM L-NAME 39.25±6.42 μm) (Supplementary Fig. 1). This finding suggests a strict role for RNS in axon degeneration.

We next explored whether SARM1 and NMNAT homologs could be potential targets for modifications by RNS in axons given that WD is prevented in cultured Sarm1^−/−^ knockout DRG neurons following exposure to the mitochondrial ROS enhancers, rotenone and paraquat^11^. For instance, tyrosine nitration involves the addition of a nitro group to the aromatic ring of tyrosine, forming 3-nitrotyrosine^54^. This modification can alter protein function, enzyme activity, and promote protein degradation^55–57^. On the other hand, tryptophan nitration involves the addition of a nitro group to the indole ring in tryptophan, affecting protein structure and function^58,59^. Both types of nitration may lead to the activation or inhibition of proteins in a context-specific manner^54,60^. To determine possible nitration sites, we investigated human SARM1 using its ligand-free crystal structure (PDB: 7LD0) in combination with DeepNitro, a database that predicts RNS modifications on amino acids^61^, and compared the predicted residues to zebrafish Sarm1 that was modeled in AlphaFold^62^. This analysis showed that human and zebrafish SARM1 had predicted nitration sites, but none of these are conserved in both species. For instance, nitration was predicted for 2 tyrosine residues and nitrosylation for two cysteine residues in zebrafish Sarm1 but in only one tyrosine residue in human SARM1 (Supplementary Fig. 2, 3). The tyrosine residue predicted to be nitrated in human SARM1 is found in a portion of the SAM^2^ domain close to the ARM domain but does not appear to be in proximity to a substrate binding site, nor does it change its position upon substrate binding with NMN when comparing the crystal structures of NMN bounded (PDB: 7NAL), or with activated SARM1 (PDB: 8GQ5) and unbounded SARM1 (Supplementary Fig. 4). If nitration occurs at this position and plays a role, the underlying mechanisms may have diverged evolutionarily in humans.

NMNAT2 is the primary NMNAT homolog shown to participate in WD due to its cytoplasmic localization^63^, but also mitochondrial NMNAT3 was shown to confer axonal protection when overexpressed^15^. Since there was no crystal structure available for human or zebrafish Nmnat2, we used the crystal structures of human NMNAT1 (PDB: 1GZU) and NMNAT3 (PDB: 1NUP) and compared these structures to human NMNAT2 and zebrafish Nmnat1/2/3 which were modeled in AlphaFold^62^. These models served as baseline for DeepNitro predictions of nitration/nitrosylation. For human NMNAT1, three amino acid residues were predicted to be nitrated, two of which are in direct proximity to the NMN substrate binding site (Fig. 3c). The residues Trp92 and Trp169 (W92/169) are located closest to the binding site whereas Tyr64 (Y64) is more distant. Interestingly, Trp169 had been previously suggested to bind to NMN^64^, and given that Trp92 is located in close proximity to this site, it may influence the binding interactions when nitrated. More intriguingly is the observation that Trp92 is predicted to be nitrated in all NMNAT isoforms and conserved between human and zebrafish (Supplementary Fig. 5), making it a likely candidate for nitration-dependent NMN substrate binding modifications. Besides potential changes in substrate interactions, Trp169 in NMNAT1 could also play a role as a protein degradation recognition site^65^, or in the adoption of a conformational change due to its superficial location on the protein surface^66^. Zebrafish Nmnat1 harbors four predicted sites for RNS modifications (Cys63, Trp91/162, and Tyr266), whereas its Nmnat2 homolog is predicted to be nitrated at Trp92 and Trp150 (Supplementary Fig. 6a, b). Human NMNAT2 only harbors Trp92 as possible nitration site, further validating its importance (Supplementary Fig. 6c). When investigating NMNAT3, we found that Tyr64 is not conserved in this human homolog, but two Trp residues (90/148) are present, which are both flanking the NMN site, similar to Trp92 and 169 in NMNAT1 (Fig. 3c, d) and DeepNitro predictions showed that these two conserved tryptophan residues are prone to nitration. Another cysteine residue at position 164 (Cys164) was predicted to undergo S-nitrosylation, which could further impact NMNAT3 function. This residue is located at the surface of NMNAT3 and may participate in similar functions as Tyr64 in NMNAT1. Comparison of this human homolog to zebrafish Nmnat3 revealed one tryptophan residue prone to nitration in zebrafish, Trp90, whereas the other residue in proximity to the substrate binding site (Trp141) was conserved but not predicted to be nitrated (Fig. 3e). Moreover, Cys164 found in human and Cys157 in zebrafish Nmnat3 was not conserved in human and zebrafish NMNAT2. Thus, the most likely residue that could alter NMN substrate binding interactions via nitration in a conserved manner is Trp90/92 in human and zebrafish NMNAT homologs.

**Fig. 4:**
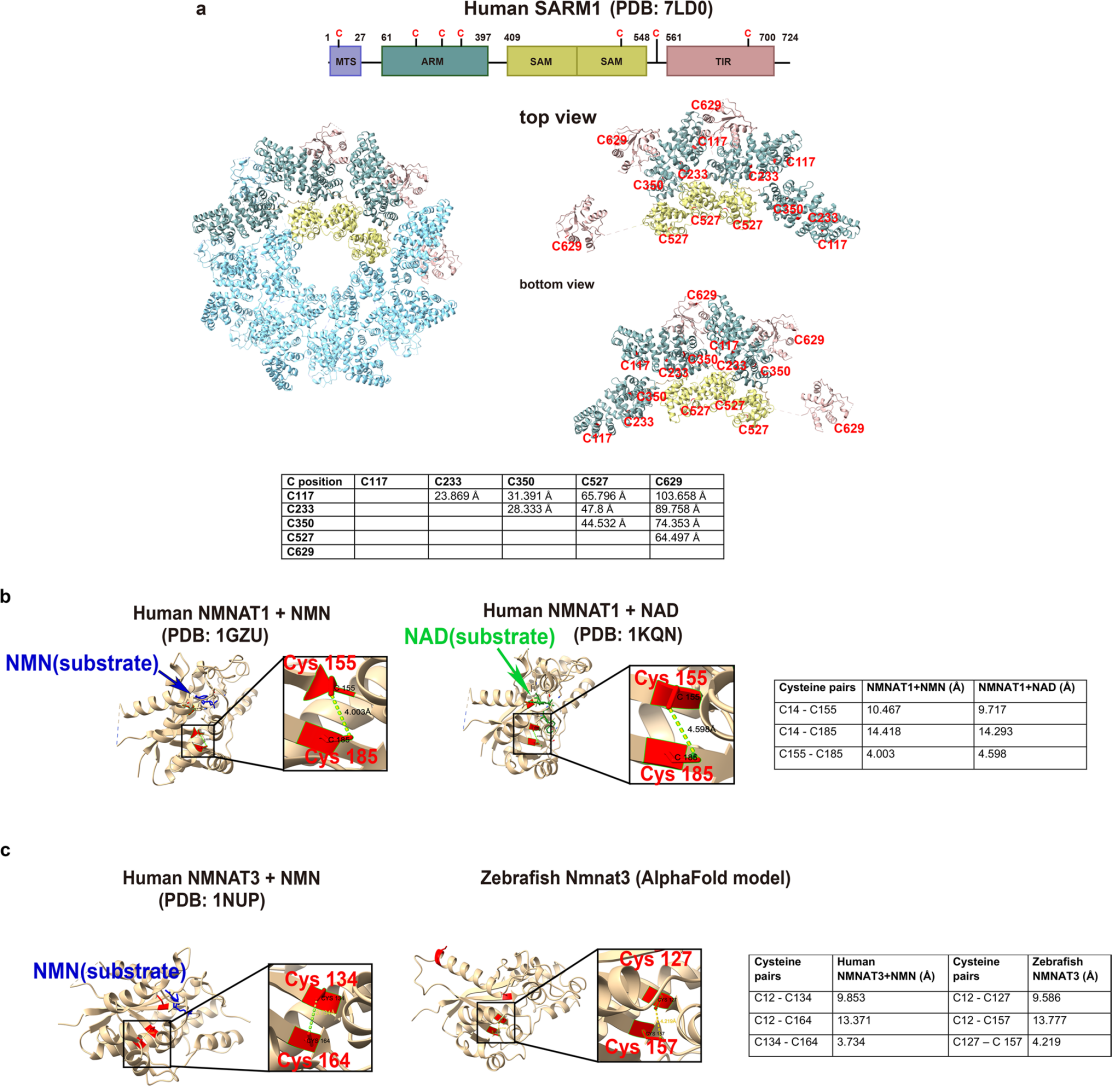
Modeling disulfide bonds in NMNAT and SARM1 shows oxidation susceptibility of NMNAT more than SARM1. (**a**) Human SARM1 octamer ligand-free crystal structure (PDB 7LD0). Conserved cysteine residues are labeled in red. The distance between each cysteine thiol group is shown in the table. (**b**) Human NMNAT crystal structure with NMN (blue) (PDB 1GZU) and NAD (green) (PDB 1KQN). Cysteines 14,155 and 185 are shown in red. Distance between C14 and C155 are shown when bound with NMN (left) and NAD (right). Squares are zoomed images and show the distance between the C155 and C185 thiol groups. Distances between all candidate cysteines are indicated in Angstrom and are shown in the table. (**c**) Human NMNAT3 compared with zebrafish Nmnat3 that was modeled in Alpha-fold due to the lack of a zebrafish crystal structure. Distances between cysteine thiol groups are indicated in the table.

Besides nitration and nitrosylation, H_2_O_2_ and peroxynitrite can also oxidize thiol groups in oxidation-sensitive cysteine residues of enzymes, leading to disulfide bond formation and structural changes that may affect the enzymatic function of proteins^42,67–69^. We therefore explored the potential of disulfide bond formation in SARM1 and NMNAT. First, we analyzed the SARM1 crystal structure in ChimeraX (https://www.cgl.ucsf.edu/chimerax/) to assess the potential of disulfide bond formation, by measuring the distances between different cysteine residues in the same protein subunit or between two subunits. Disulfide bonds are typically formed when the thiol groups are in proximities below ~ 2.2Å (PDB database cutoff: >3.0Å)^70^. Seven conserved cysteine residues were identified in human SARM1 and zebrafish Sarm1, however, 3D modeling in ChimeraX revealed that under the above criteria, none appeared to be close enough to form disulfide bonds under oxidative conditions (Fig. 4a, Supplementary Fig. 7). These results fit the suggested model that SARM1 activation is primarily determined by NMN substrate binding^71^.

We next predicted the potential for cysteine oxidation in human and zebrafish NMNAT1/2/3. Using the crystal structure of NMNAT1 bound with NMN (PDB:1GZU), we examined the spatial relationship between conserved cysteine residues and the NMN binding site, which involves Ser16, Thr95, and Trp169. We identified two conserved cysteine residues (Cys155 and Cys185) with a measured distance of ~ 4.003Å at a resolution of 2.9Å. Given the resolution limitations and possible conformational shifts under different conditions, these residues could potentially form a disulfide bond. Both cysteine residues are also conserved in zebrafish Nmnat1 (Cys148/178) but their distance is predicted to be further apart (4.68Å) (Supplementary Fig. 5, 8). The zebrafish prediction, however, does not consider NMN binding, which was used in the prediction of human NMNAT1.

The distance between a third cysteine residue Cys14 in human NMNAT1 and Cys155/Cys185 each exceeded 10Å, making these unlikely to form disulfide bonds. Analysis of another crystal structure of NMNAT1 bound to NAD^+^ (PDB: 1KQN), further increased the distance between Cys155 and Cys185 to 4.59Å (at a resolution of 2.2Å) (Fig. 4b). Considering that the protein structure can be influenced by factors such as pressure, temperature, and pH, leading to conformational variations, it is possible that this disulfide bond may also form under certain conditions with substrate, which remains to be experimentally determined. Since Cys155 and Cys185 are in the inner core of the protein and close to the substrate binding site, a disulfide bond, if formed, could affect NMNAT/NMN substrate binding. In this scenario, oxidation may lead to the release of NMN or tighter binding of NAD^+^. There we no predicted cysteine residues in either zebrafish or human NMNAT2, which are close enough to form disulfide bonds (Supplementary Fig. 9).

Examination of disulfide bond formation in NMNAT3 using the human crystal structure of NMNAT3 bound with NMN (PDB: 1NUP) identified 3 cysteine residues that are also conserved in zebrafish NMNAT3 (human: Cys12, 134, and 164; zebrafish: Cys12, 127, and 157). Of these, Cys134/164 in human NMNAT3 and Cys127/157 in zebrafish Nmnat3 showed distances of 3.7Å and 4.2Å, respectively (Fig. 4c), indicating the potential for disulfide bond formation when correcting for resolution and potential changes in crystallization conditions. These findings suggest the possible redox regulation of NMNATs besides nitration, which could contribute to NMNAT inactivation, which remains to be experimentally tested. Oxidative regulation may however be specific to NMNAT1 and NMNAT3 since NMNAT2 does not harbor cysteine residues which are predicted to be oxidized (Supplementary Fig. 4).

### NADPH treatment reduces sensory axon degeneration after skin injury

**Fig. 5:**
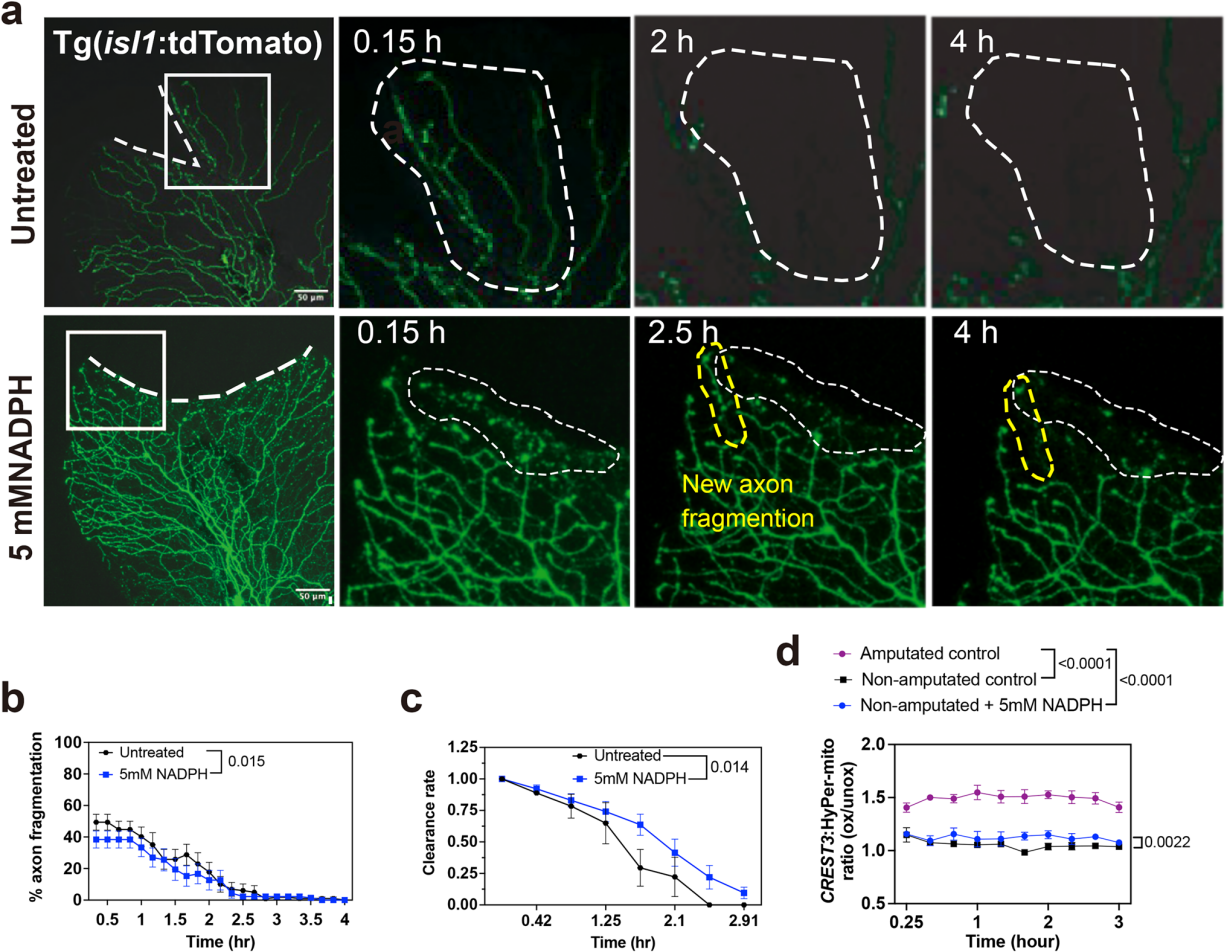
NADPH treatment delays axon degeneration. (**a**) Time-lapse images showing axon fragmentation following amputation for untreated, and 5 mM NADPH treatment. Dashed line in first image of each treatment group outlines the amputation plane. White circular dashes represent the area of axon fragmentation. Yellow circles outline areas of new fragmentation. (**b**) Percentage of axons that fragment over the course of 4 h (untreated: *n* = 13, 5mM NADPH: *n* = 12). (**c**) Fluorescence intensity of axon fragments over time in the same fish shows delayed clearance with NADPH compared with untreated fish. (**d**) CREST3:HyPer-mito expression in sensory neurons showing significantly elevated mito-H_2_O_2_ levels in axons of NADPH-treated fish but significantly less than those produced following amputation (Non-amputated control: *n* = 6, Amputated control: *n* = 5, Non-amputated + 5mM NADPH: *n* = 4). (Two-way ANOVA and Tukey’s post-test).

Our previous work showed that axon degeneration following fin amputation is significantly delayed in zebrafish *cyba*^−/−^ mutants with a non-functional P22phox subunit that activates all NADPH oxidase enzymes except Nox5^40^ which is not expressed in larval zebrafish^3^. Since NADPH is not utilized by Nox enzymes in these mutants, we hypothesized that this electron carrier may delay axon degeneration through its redox activity, which could be therapeutically beneficial for treating neurodegenerative diseases. To test this, we pharmacologically treated wildtype larval zebrafish at 3dpf with NADPH to assess its effects on axon degeneration. We initially determined the MTD for NADPH when treating Tg(*isl1*:tdTomato) zebrafish, which showed that 5mM and 1mM NADPH did not affect the survival of amputated zebrafish larvae. The experiments were therefore conducted using the 5mM NADPH concentration. Treatments at 3dpf started 30 min prior to amputation for 4 h during which time-lapse imaging was conducted. This showed that NADPH treatment decreased the percentage of axons undergoing fragmentation by about 10%, which was small but significant (Fig. 5a, b). Prolonged treatment promoted delayed fragmentation onset beyond 2hpa. Interestingly, the clearance rate of axon fragments was slower compared with untreated controls (Fig. 5a, c) and NADPH had a small but significant effect on axonal mitochondrial H_2_O_2_ levels, which were slightly increased compared with untreated controls, although this was significantly less than the increase seen following amputation (Fig. 5d). Potentially, NADPH dependent mitoH_2_O_2_ production could have stabilizing effects on axons leading to delayed fragmentation, consistent with a known role for H_2_O_2_ in axon regeneration^1^.

**Fig. 6:**
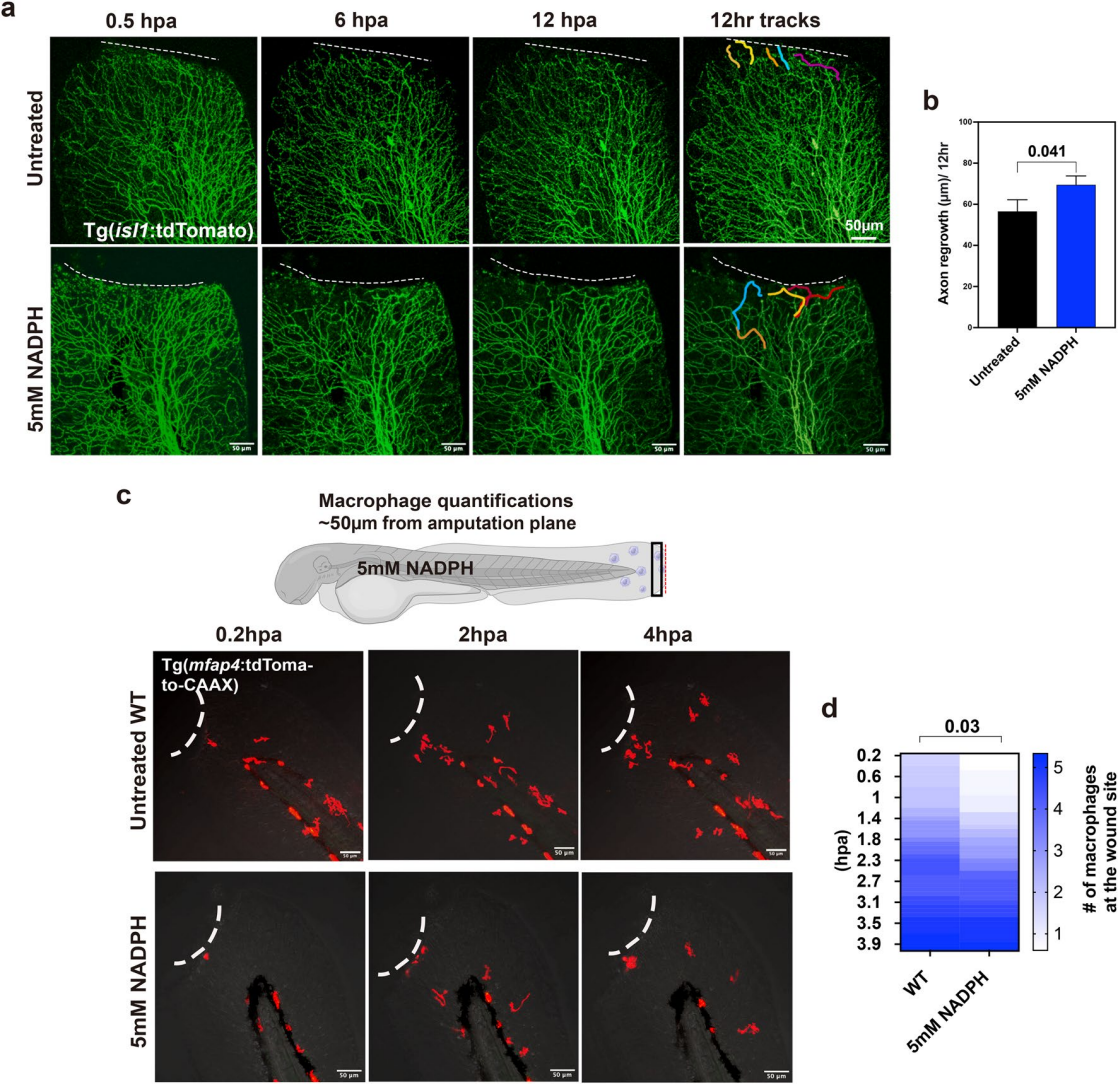
NADPH treatment promotes axon regeneration. (**a**) Maximum intensity projections of 12 h time-lapse recordings following amputation shows enhanced axon regeneration when fish are treated with 5mM NADPH compared with untreated wildtype controls. Dashed lines outline the amputation plane. The rightmost panel shows the growth cone trajectories (each color indicates one axon branch). (**b**) Comparison of axon growth in wildtype and 5mM NADPH treated fish indicates a significant enhancement of regeneration in the NADPH-treated fish (*n* = 6 per group, Student’s T-test). (**c**) Tg(*mfap4*:tdTomato-CAAX) fish at 3dpf following amputation shows a continuous reduction in macrophages at the wound. (**d**) Quantification of macrophages at the wound shows a delay by 1–2 h following amputation compared with amputated untreated wildtype fish (untreated: *n* = 6, 5mM NADPH *n* = 5, Student’s t-test).

The observation that NADPH reduced axon degeneration and clearance of axon fragments following injury prompted us to ask whether NADPH treatment also promotes cutaneous sensory axon regeneration, which begins at approximately 2hpa^1,32^. To assess regeneration, we treated Tg(*isl1*:tdTomato) fish with 5 mM NADPH starting 30 min prior to amputation and tracked axon growth until 12pa using time-lapse imaging. Compared with wildtype, axons, regeneration was significantly increased in NADPH-treated fish (Untreated: 56.56±5.63 μm vs. 5mM NADPH: 69.48±4.31 μm, SEM) (Fig. 6a, b). Together, this shows that NADPH decreases axon degeneration and promotes axon regeneration, and that increased H_2_O_2_ levels in axonal mitochondria may be growth promoting while also influencing degeneration dynamics.

The clearance of axon fragments following laser axotomy in larval zebrafish has been shown to be mediated by both keratinocytes and macrophages^72^. Since clearance was delayed in NADPH treated fish, we analyzed whether this effect could be related to delayed migration of macrophages to the wound. Indeed, the macrophage number until ~ 2.5hpa was significantly reduced compared with wildtype control fish but was comparable thereafter (Fig. 6c, d). The clearance of axon fragments could thus be mediated by a delayed arrival of macrophages at the wound during the initial degeneration phase. NADPH could potentially counteract the ROS gradient that is generated at the amputation wound and which was shown to be important for macrophage migration toward the wound^3^. Alternatively, it may impact redox related fragmentation processes leading to the engulfment of debris^73^.

### NADPH rescues paclitaxel-induced peripheral neuropathy

**Fig. 7:**
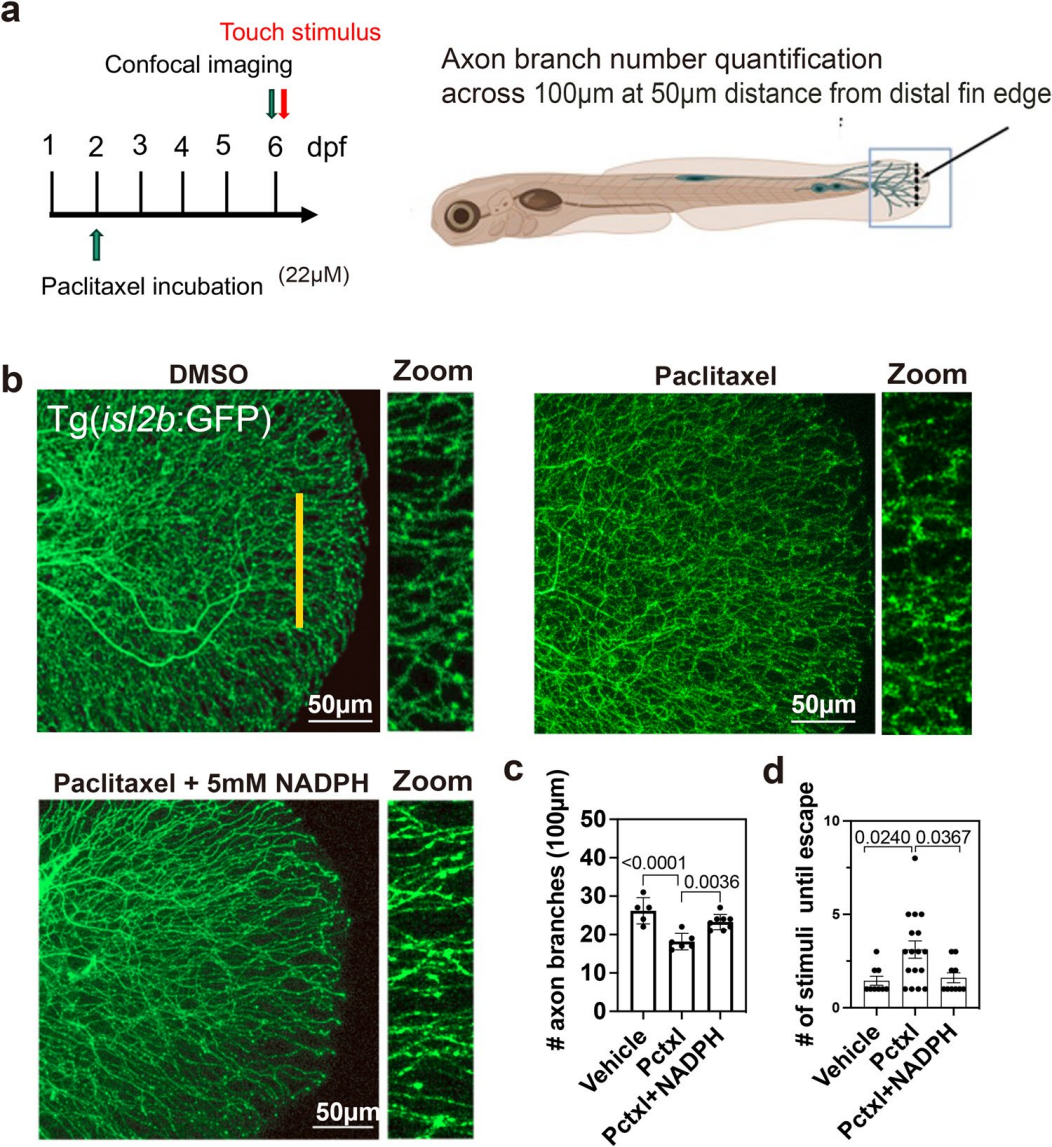
NADPH prevents paclitaxel neurotoxicity. (**a**) Schematic showing experiments in a-c. (**b**) Axon branches in the caudal fin of Tg(*isl2b*:GFP) fish. The yellow line marks the region in which the number of axons was quantified for each fish (line width = 100µM and 50 μm from the distal fin edge). (**c**) Comparison of the number of axon branches in vehicle, 22µM paclitaxel, and paclitaxel + 5mM NADPH treated fish at 6dpf (4-day treatment) shows a significantly reduced axon branch number in paclitaxel-treated compared with vehicle and paclitaxel + 5mM NADPH treated fish (vehicle *n* = 5, paclitaxel *n* = 6, paclitaxel + 5mM NADPH *n* = 8, one-way ANOVA). (**d**) Quantification of the number of touches necessary to elicit an escape response following 4 days of treatment shows a significant increase in paclitaxel-treated but not vehicle and pactlitaxel + 5mM NADPH treated fish (vehicle *n* = 9, paclitaxel *n* = 17, paclitaxel + NADPH *n* = 9, one-way ANOVA).

The beneficial effects of NADPH on injury-induced axon degeneration suggested that this electron carrier may be therapeutically interesting. We therefore analyzed its effects in a zebrafish model of chemotherapy-induced peripheral neuropathy (CIPN). We previously developed this model in zebrafish using the chemotherapeutic agent paclitaxel, which was shown to cause the degeneration of unmyelinated cutaneous sensory axons in the epidermis^74–77^. We showed that paclitaxel promotes the dying back of cutaneous sensory axons from the caudal fin edge, correlating with a diminished tactile response^75^. To monitor axon behavior in the presence of NADPH, we used zebrafish Tg(*isl2b*:GFP) fish in which all sensory neurons are fluorescently labeled. These fish were incubated for 96 h from 2-6dpf with vehicle, 22µM paclitaxel and 22µM paclitaxel + 5mM NADPH. Toxicity was monitored by assessing the tactile response to touch with a capped microloader pipette tip and by quantification of the axon branch number from confocal images recorded at 6dpf (Fig. 7a). Compared with paclitaxel-treated fish, which significantly decreased the axon branch number, vehicle and co-administration of 5mM NADPH with paclitaxel significantly prevented this decrease (Branch number along 100 μm: vehicle: 26.2±1.53 vs. paclitaxel: 18.17±0.87 vs. paclitaxel + NADPH: 23.25±0.7, SEM) (Fig. 7b, c). The tactile response assay showed that paclitaxel + NADPH treatment prevented the delayed response to touch observed in fish treated with paclitaxel alone (vehicle: 1.44±0.24 vs. paclitaxel: 3.12±0.46 vs. paclitaxel + NADPH: 1.6±0.27, SEM) (Fig. 7d). Thus, NADPH prevents axon degeneration and paclitaxel-induced numbness likely through mechanisms that change the redox balance inside cells.

## Discussion

Our study provides novel insights into the interplay between RNS and ROS in sensory axon degeneration and regeneration following zebrafish fin amputation. We demonstrate that RNS, particularly peroxynitrite (ONOO⁻), are primary drivers of axon degeneration, whereas ROS, specifically H₂O₂, promote regeneration, likely in part through epidermal pathways. Additional antioxidant enzymes, including catalase, may also influence H₂O₂ dynamics in this process, but their role remains to be determined. These findings underscore the complex balance between oxidative stress and neuronal repair mechanisms, suggesting potential therapeutic strategies for neurodegenerative diseases.

A key discovery in our study is the differential role of ROS and RNS in sensory axon fate following injury. While previous studies have implicated ROS, particularly H₂O₂, in axon regeneration^1,40^, our results highlight the degenerative role of RNS, particularly peroxynitrite (ONOO⁻). These findings align with evidence from neurodegenerative diseases where superoxide and nitric oxide contribute to peroxynitrite formation, leading to DNA oxidation^78^, lipid peroxidation^79^, and protein oxidation and nitration^58^. In conditions such as Alzheimer’s and Parkinson’s disease, for instance, peroxynitrite-induced oxidative damage of proteins, lipids, and DNA has been linked to neuronal dysfunction and cell death^25^, and peroxynitrite accumulation correlating with Parkinson’s disease severity^27^.

In contrast, various studies, including our own, suggest that the ROS, H₂O₂, facilitates axon regeneration, often through neuron-extrinsic pathways^80–82^. These studies indicate that ROS/RNS not only differ in their function but also in the types of tissues and cell types they affect. We previously showed that H_2_O_2_ promotes axon regeneration following zebrafish fin amputation through its effects on epidermal keratinocytes, leading to Epidermal Growth Factor Receptor (EGFR) activation and extracellular remodeling via Matrix-Metalloproteinase 13 (MMP-13), thereby providing a growth path for regenerating axons^40^. In this study, and consistent with previous findings, the role of superoxide and RNS in axon degeneration appears to be neuron intrinsic. Our findings suggest that H_2_O_2_ may function by remodeling the environment to promote axon growth whereas axonal mitochondrial RNS stimulate axon degeneration, possibly through nitration/nitrosylation of downstream factors, such as NMNAT.

Axon degeneration has been shown to follow a molecularly defined pathway, known as Wallerian degeneration, involving two major players, NMNAT and SARM1^12,14,83–89^. Upon injury, NMNAT is degraded, leading to the accumulation of its substrate, NMN, and the subsequent activation of SARM1 which depletes NAD⁺. This sets in motion a chain of events^22,85^ leading to the degeneration of the distal axon fragment that was separated from the cell body during the injury^90^. WD has been shown to depend on ROS, as DRG neurons from SARM1 knockout mice treated with the mitoROS inducers, paraquat and rotenone, do not degenerate, unlike wildtype control DRGs treated with these compounds^11^. Thus, SARM1 appears to act downstream of mitoROS in this pathway. Our analysis suggests however that RNS modifications are more likely to occur in NMNAT than SARM1. While we did not find convincing evidence that SARM1 is modified by either oxidation or nitration/nitrosylation, two potential modifications in NMNAT may occur and influence its regulation: (1) tryptophan nitration and (2) cysteine oxidation (restricted to human and zebrafish NMNAT1 and NMNAT3). Modifications by nitration could participate in the regulation of NMN substrate binding, or in the exposure of the protein to post-translational modifications such as ubiquitination, leading to protein degradation^91^. While the predicted cysteine residues in NMNAT1 and NMNAT3 may theoretically form disulfide bonds, the absence of these critical cysteines in NMNAT2 suggests that cysteine oxidation dependent regulation is not central to all NMNAT activity and may be restricted to particular cellular compartments, such as the nucleus (NMNAT1) and mitochondria (NMNAT3) where oxidation is prominent^92^. Nitration of other tryptophan residues in various NMNAT isoforms could play other significant roles. For instance, nitration of tryptophan residue 148 in human NMNAT3 and 169 in zebrafish Nmnat3 might disrupt NMN binding by altering the indole ring, impairing NAD⁺ biosynthesis^64^. Similarly, nitration of tryptophan residue 90/92 in all isoforms of both human and zebrafish NMNAT, which is located near the NMN substrate-binding site, may reduce the affinity for NMN binding and alter the enzymatic activity. The conservation of this tryptophan underscores its functional significance. To better understand the mechanism involving nitration, nitrosylation, and oxidation, biochemical studies are needed to examine these modifications in more detail. For example, antibody staining and mass spectrometry combined with site-directed mutagenesis could identify specific nitration events and their impact on NMNAT structure and function.

In this study we chose GPx1a as our H₂O₂-scavenging enzyme because of its dual cytosolic and mitochondrial localization^93^, which is relevant to the mito-H₂O₂ signals measured in vivo. GPx1a activity directly couples H₂O₂ detoxification to the glutathione redox cycle. Catalase, in contrast, is predominantly peroxisomal^45,94,95^ and may therefore play a lesser role in mitochondrial or cytosolic H₂O₂ clearance in somatosensory neurons, although it could still influence extracellular H₂O₂ gradients via non-neuronal cells. Future work will be needed to assess the contributions of other antioxidant components, including catalase, and to determine how these enzymes influence axon de- and regeneration, and ROS/RNS formation.

We used the NADPH treatment to test its therapeutic potential and demonstrated with Hyper ratiometric imaging an increase in H_2_O_2_ formation following NADPH treatment, unlike expected if NADPH had antioxidant functions and these prevent degeneration. Our data instead suggests that exogenous NADPH may have served as a substrate for NADPH oxidases to increase mitochondrial H_2_O_2_ production in sensory neurons, consistent with its substrate functions. This increase, which was significantly below the levels measured following amputation, may have contributed to the pro-regenerative effects observed with NADPH treatment. However, NADPH may also have played a role in protein regulation, promoting regeneration and maintaining axonal homeostasis, and this contributed to the reduction in axon fragmentation. How can this dual role be explained? A likely possibility is that the observed effects are cell type specific, which we did not assess in our in vivo studies using pharmacological treatments. For instance, NADPH is essential for satellite glial cell elongation to support axon regeneration in the peripheral nervous system whereas in neurons, NADPH participates in the transfer of its hydrogen to NAD^+^, converting it into NADH, which diffuses into mitochondria for participation in ATP production, and this directly helps support axon regeneration while inhibiting axon degeneration^96^. Another effect of elevating NADPH levels in our study via pharmacological means may have been an increase in H_2_O_2_ in the skin. This may have positively influenced axon regeneration via EGFR/MMP-13 dependent extracellular matrix remodeling^40^. Further in vivo studies are needed to analyze the involvement of antioxidant systems in the context of NADPH treatment and to better understand the cell type specific roles of NADPH and its neurotherapeutic potential in delaying axon degeneration and/or promoting regeneration.

## Materials and methods

### Ethical approval

All experiments were performed in accordance with guidelines and regulations approved by the University of Miami’s Institutional Animal Care and Use Committee (IACUC) (# A-3224-01) with accreditation from the Association for Assessment and Accreditation of Laboratory Animal Care (AALAC) (# 001069). Live vertebrate studies adhered to the ARRIVE Guidelines and the Guide for the Care and Use of Laboratory Animals. The ethical implications of using zebrafish were carefully considered and weighed against alternative methods. The unique optical clarity of zebrafish allows for non-invasive, real-time imaging of cellular and molecular processes that are not reproducible using computational modeling or cell culture. Thus, the scientific and societal benefits derived from this research justify the ethical considerations.

### Zebrafish husbandry

Wild-type zebrafish (AB and Tuebingen strains) were purchased from the Zebrafish International Resource Centre (ZIRC). Zebrafish eggs were collected with a strainer and rinsed with deionized water, then placed into Petri dishes with Ocean medium along with Methylene Blue. Dead embryos were removed the next day and healthy eggs placed into Ringer’s solution. Phenylthiourea (PTU) was added starting at 1dpf to prevent pigment formation. Zebrafish were incubated at 28.5 °C in a 14:10-hour light: dark cycle until experimental start.

### Transgenic lines

The transgenic lines Tg (*isl1*:lexA-lexAop_tdTomato) and Tg(*isl2b*:GFP) were utilized to visualize somatosensory neurons. Tg(*mfap4*:tdTomato-CAAX) transgenic fish were used to assess macrophages at the wound.

### Cloning

Crest3:Gal4VP16:dsRed_5xUAS_SOD2: pDONR vectors for CREST3 and Gal4VP16-14xUAS were a gift from Dr. Alvaro Sagasti (UCLA). For cloning of SOD2 cDNA, total RNA from 3 dpf zebrafish of AB fish line was isolated using RNeasy kit (Qiagen, Cat. No. 74004); and reverse transcript into cDNA using reverse transcriptase (SuperScript IV Reverse Transcriptase, Invitrogen, Cat. No. 18090010). Fragments were amplified with PCR primers: SOD2 Forward, 5’ CCTCGAAGACGGTCGCTTGTATCACTGTG 3’,

SOD2 Reverse, 5’ TATCATGTCTGGATCATCATGCGCTTCGAAAGTACAAAAAAG 3’,

Crest3 Forward, 5’ AAACACAGGCCAGATGGGCCGTATAGAAAAGTTGGTAACAGGATGTG 3’,

Crest3 Reverse, 5’ GTAGCTTCATAAACTTGGCCTGCTGCTG 3’,

Gal4VP16 Forward, 5’ GGCCAAGTTTATGAAGCTACTGTCTTCTATC 3’,

Gal4VP16 Reverse, 5’ AGGAGGCCATGCAGTGAAAAAAATGCTTTATTTG 3’,

DsRed2 Forward, 5’ TTTCACTGCATGGCCTCCTCCGAGAAC 3’,

DsRed2 Reverse, 5’ ACCCTCTAGACTACAGGAACAGGTGGTG 3’,

5XUAS Forward, 5’ GTTCCTGTAGTCTAGAGGGTATATAATGGATCCCATC 3’.

5XUAS Reverse, 5’ ACAAGCGACCGTCTTCGAGGTCGAGGGAATTC 3’.

pDEST vector was digested with Cla1 and Apa1 enzymes. The fragments (CREST3, Gal4VP16, dsRED, 5xUAS, and SOD2) were subsequently recombined with the pDEST vector.

Crest3:Gal4VP16_5xUAS_HyPer-mito:

The expression vector was assembled using pDONR vectors for CREST3 and Gal4VP16-14xUAS (gift of Dr. Alvaro Sagasti, UCLA). First, a HyPer-mito fragment was amplified from pHyPer-dMito (Evrogen, Cat. No. FP942) using PCR using the following primers:

pHyper-dMito: Forward: 5’ TATACCCTCTAGAGGTTTAGTGAACCGTCAG 3’,

pHyper-dMito: Reverse: 5’ TATCTTATCATGTCTCCATCATCATTAAGATACATTGATGAGTTTGG 3’,

Crest3 Forward, 5’ GTCTGAAACACAGGCCAGATGGGCCGTATAGAAAAGTTGGTAACAGGATGTG 3’,

Crest3 Reverse, 5’ CAGTAGCTTCATAAACTTGGCCTGCTGCTG 3’,

Gal4VP16 Forward, 5’ GCAGGCCAAGTTTATGAAGCTACTGTCTTCTATC 3’,

Gal4VP16 Reverse, 5’ GACCTCGAAGAGCAGTGAAAAAAATGCTTTATTTG 3’,

5XUAS Forward, 5’ TTTTTTTCACTGCGTCTTCGAGGTCGAGGGAATTC 3’,

5XUAS Reverse, 5’ GTTCACTAAACCTCTAGAGGGTATATAATGGATCCCATC 3’.

The pDEST vector was subsequently digested with ClaI and ApaI and recombined with CREST3, Gal4VP16, 5xUAS, and HyPer-mito.

### Plasmid injections

Fertilized eggs at the early 1-cell stage from a wildtype AB strain were injected with ∼25pg transgene plasmid using a micropipette injection system (ASI Instruments, USA) and the plasmids as indicated in the text. Injected and transgenic embryos were grown to their respective ages as indicated in the text in a 28.5 °C incubator, which was set to a 14:10 h light: dark cycle. Crest: Gal4VP16:5XUAS: gpx1a was previously published^40^. For transient, mosaic expression in sensory neurons, ∼25pg of each transgene plasmid was injected into newly fertilized wildtype AB fish.

### Zebrafish drug treatments

Embryos were dechorionated with 1 mg/ml Pronase (Millipore Sigma, Cat. No. 10165921001) or forceps and anesthetized in 1:1,000 2-Phenoxyethanol ((Millipore Sigma,77699). Paraquat was initially maintained as 25mM stock solution in DMSO and then further diluted to 10µM in Ringers for fish treatment. Rotenone was stored at -20 C as a stock concentration of 10mM in DMSO and diluted to 0.1µM in Ringers for experimental use. Control solutions were prepared by adding equal volumes of DMSO (vehicle) to Ringer’s solution. Peroxynitrite concentrations of 10µM were diluted in Ringer’s solution prior to the experiment. NADPH was diluted in Ringer’s solution to concentrations of 5mM for experimental use. Larvae were incubated in the indicated concentrations for 30 min before and after fin amputation to assess axon degeneration and axon growth activity. 10µM of L-NAME was diluted in Ringer for use, and larvae were incubated in the indicated concentrations for 3 h before and after fin amputation to assess axon degeneration and axon growth activity.

For paclitaxel treatments between 2 and 6dpf, a 5.8mM paclitaxel stock solution in DMSO was prepared and kept at -20 °C. The stock was diluted to 22µM paclitaxel in Ringer’s solution prior to the experiment. Control solutions contained 0.05% DMSO vehicle. For axon degeneration and regeneration studies, the larvae were incubated in various treatment solutions (as mentioned in main text) at indicated concentrations for 3 h prior to imaging and maintained in the solution during the entire imaging period. A 1:1,000 dilution of 2-phenoxyethanol was added during imaging to maintain the fish anesthetized. The fish were euthanized in a 1:500 2-phenoxyethanol/Ringer’s solution following completion of imaging.

### Larval zebrafish fin amputation

Larval fish were anesthetized for 10 min in a 1:1,000 dilution of 2-phenoxyethanol and mounted in a 1% low-melt agarose in an imaging chamber. The fin was severed under a stereomicroscope using a syringe needle and immediately transferred to a confocal microscope for imaging. Because of the hard surface, some amputations were uneven, but this did not influence axon behavior. Imaging began about 5 min post amputation.

### Larval touch response assay

At 6dpf, treated zebrafish larvae were individually transferred into a Petri dish filled with Ringer’s solution ad maintained in the solution for 15 min prior to stimulation to adopt the fish to their new environment. The tail fin was stimulated with a micro loader pipette tip until an escape response was elicited up to 10 times. The number of stimuli was quantified and graphed comparing control and paclitaxel-treated fish.

### Zebrafish imaging

Zebrafish larvae were mounted in 1% low melting agarose on glass-bottom Petri dishes. The position and orientation were adjusted using a capped microloader pipette tip (Eppendorf). For time-lapse imaging, 5–10 larvae were mounted in the same dish per session. The fish were imaged on a Zeiss LSM880 Airy Scan confocal microscope for either 4–12 h using a 10x or 20x objective. Z-stacks were set to 2.5 μm intervals. Time intervals were 10–15 min per stack. The resulting raw data was processed and analyzed in Imaris and Fiji. Z-stack images were projected into single images as maximum intensity projections for publication.

### Quantifications

Axon degeneration. Axon degeneration was assessed over time by counting the number of axons undergoing fragmentation. The percentage of axon fragmentation was determined by calculating the ratio of fragmenting axon branches to the total number of existing axon branches.

Clearance of axon debris. Axon debris clearance was assessed for each severed branch by measuring the intensity from the onset of fragmentation to the disappearance of the final fragment. The intensity ratio was determined by dividing the intensity at a specific time point (timepoint x) by the intensity at the first time point, then multiplied by 100 to calculate the percentage of axon debris.

Axon regeneration measurement. The regrowth of axon branch tips following amputation was determined by first aligning the time-lapse images using the StackReg/TurboReg plugins in Fiji. This was followed by tracking axon branch tips using the Manual Tracking tool and measuring the axon growth length.

HyPer fluorescence. To detect the presence of H_2_O_2_ via ratiometric HyPer imaging and quantification of the fluorescence ratio, images were first background-subtracted in Fiji, and fluorescent cell intensities in each channel were measured. The 488/405 ratios were calculated in Excel. Images for visualization of HyPer fluorescence were generated in the Zeiss ZEN software using the Image Calculator “Division” function to divide 488/405 channels, followed by the Eq. 488^2/(1.3*405) to visualize the fluorescence.

Macrophage Quantification. Macrophages were imaged for 4 h in amputated Tg(*mfap4*:tdTomato-CAAX) 3pf zebrafish larvae. Newly arrived macrophages at the wound site were counted during this time and compared between the control and treatment groups.

### Sequence alignments

The sequences of human (*Homo sapiens*) and zebrafish (*Danio rerio*) SARM1 and NMNAT were acquired from the NCBI protein database. Different alignments were produced in EMBL-EBI Job Dispatcher sequence analysis tools^97^.

### Protein modeling

*AlphaFold*: Human NMNAT2 and zebrafish SARM1 and NMNAT1,2,3 protein structures were generated using AlphaFold2 (ColabFold v1.5.5: AlphaFold2 using MMseqs2), which is an artificial intelligence engineered to predict protein structures based on known homologous sequences. Human SARM1 and NMNAT1/3 protein structures bounded to their substrates were obtained as PDB file for further visualization and analysis in ChimeraX. Human SARM1 (https://www.rcsb.org/structure/7LD0), human NMNAT1 with NMN (https://www.rcsb.org/structure/1GZU), human NMNAT1 with NAD (https://www.rcsb.org/structure/1KQN), and human NMNAT3 with NMN (https://www.rcsb.org/structure/1NUP).

*DeepNitro* was used for modeling of RNS modifications. Human and zebrafish SARM1 and NMNAT protein sequences (either as PDB or AlphaFold files) were used for prediction of S-nitrosylation, Tyr-nitration, and Trp-nitration. Thresholds to control the sensitivity of peak detection in nitration site predictions and determine the confidence level or probability cut-off at which a residue is classified as nitrated or non-nitrated were set to medium for RNS modification predictions.

For disulfide bond formation potential, *ChimeraX* was used. First, the PDB files were imported and visualized to determine the distances between cysteine residues by selecting two thiol group sulfur atoms and use the “distance” feature in the software to measure distances between the residues.

### Statistical analyses

Statistical comparisons were made using Prism 8 (GraphPad). The unpaired Student’s t-test with a 95% confidence interval was used to compare the means of two unmatched groups. For multiple comparison, tests of three or more groups, one or two-way ANOVA at an alpha = 0.05 (95% confidence interval) and Tukey’s multiple comparison posttest were used to compare the means of each column. Significance is denoted with asterisks: *P < 0.05, **P < 0.01, ***P < 0.001, ****P < 0.0001. The SEM is shown for all experiments where at least two biological replicates were used per group.

## Supplementary Information

Below is the link to the electronic supplementary material.

Supplementary Material 1

## Data Availability

The predicted zebrafish Nmnat3 structure and its potential post-translational modifications were analyzed using AlphaFold and the data was deposited in the ModelArchive repository (https://modelarchive.org/doi/10.5452/ma-2xaqv). Previously deposited reference structures include human NMNAT2 (Q9BZQ), zebrafish Sarm1 (F1QWA8), zebrafish Nmnat1 (E9QH56), and zebrafish Nmnat2 (Q6PC93).
