# Supplementary material for: A redox-dependent switch governing sensory axon degeneration and regeneration
Source: Sci Rep. 2025 Nov 11;15:39434. doi: 10.1038/s41598-025-23035-6 (PMC12606303; doi:10.1038/s41598-025-23035-6)
Supplement: Supplementary file 1 — Supplementary Material 1 [file 41598_2025_23035_MOESM1_ESM.pdf]

**Title: A Redox-Dependent Switch Governing Sensory Axon Degeneration and Regeneration**

**Authors:** Chia-Jung Hsieh[1], Lauryn Lee[1] and Sandra Rieger[1,2]

**Affiliations**:

[1] Department of Biology, University of Miami, Coral Gables, FL 33146
[2]Sylvester Comprehensive Cancer Center, Miller School of Medicine, University of Miami, Miami, FL 33136

**Supplemental Figures & Legends**

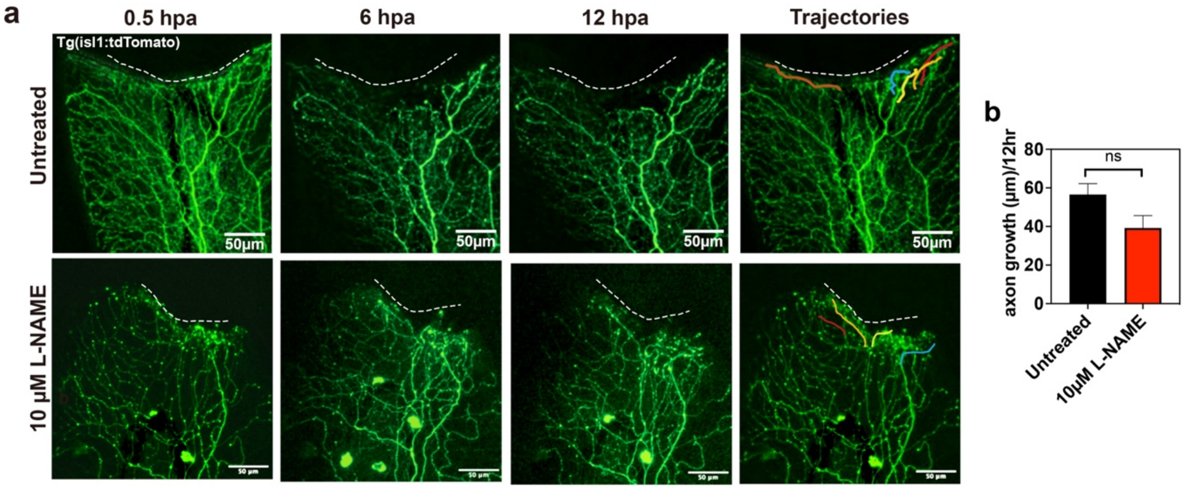


**Figure S1. Axon regeneration is not significantly impacted in the absence of RNS.** (**a**) Time-lapse images of axon regeneration over 12 hours. The rightmost panels show axon trajectories over time (each color indicates one axon branch). (**b**) Comparison of axon growth in untreated fish and L-NAME treated fish shows no significant difference (Unpaired Student's t-test, $p>0.05$).

```
hsSARM1 MVLTLLLSAYKLCRFFAMSGPRPGAERLAVPGPDGGGGTGPWWAAGGRGPREVSPGAGTE 60
drSarm1 MFLSLVVYLSKICRYLSMFSSD----RLTVPEYVSSR---LHNRRTAPDPRAVSPGISTD 53

hsSARM1 VQDALERALPELQQALSALK---QAGGARAVGAGLAEVFQLVEEAWLLPAVGREVAQGLC 117
drSarm1 VQAVLDGSLPALRSAIRTLRSSKDTGDLEETRRAIAETFQLVEEAWVLPTVGRRVAEEIC 113

hsSARM1 DAIRLDGGLDLLLRLLQAPELETRVQAARLLEQILVAENRDRVARIGLGVILNLAKEREP 177
drSarm1 NRIRLDGGLELLLQLMQTPAVEITYESAKLLEQILVSENRDYVARMGLGVILNLTREQED 173

hsSARM1 VELARSVAGILEHMFKHSEETCQRLVAAGGLDAVLYWCRRTDPALLRHCALALGNCALHG 237
drSarm1 AQLARSVSGILEHMFKHTEETSAQLITNGALDTILYWCRGTDPTVLRHCAVALSNCAMYG 233

hsSARM1 GQAVQRRMVEKRAAEWLFPLAFSKEDELLRLHACLAVAVLATNKEVEREVERSGTLALVE 297
drSarm1 GHRCQRLMIEKQAAEWLFPLAFSKEDELIRFHACLAVAVLAANREMEKEVVKSGTLELVE 293

hsSARM1 PLVASLDPGRFARCLVDASDTSQGRGPDDLQRLVPLLDSNRLEAQCIGAFYLCAEAAIKS 357
drSarm1 PFIASLDPDEFARNMLDSADSMQGRTAADLQHLLPLLDGTRLEGKCIAAFYLCVETSIKS 353

hsSARM1 LQGKTKVFSDIGAIQSLKRLVSYSTNGTKSALAKRALRLLGEEVPRPILPSVPSWKEAEV 417
drSarm1 RQRNTKIFQEIGAVQSLKRIVMYSSNATVCSLAKRALKMMSEEVPRRILSSVPNWKSGEV 413

hsSARM1 QTWLQQIGFSKYCESFREQQVDGDLLLRLTEEELQTDLGMKSGITRKRFFRELTELKTFA 477
drSarm1 QTWLQQIGFSAFSERFQELQVDGDLLLNITEQDLIQDLGMTSGLTRKRFLRDLRVLKTYA 473

hsSARM1 NYSTCDRSNLADWLGSLDPRFRQYTYGLVSCGLDRSLLHRVSEQQLLEDCGIHLGVHRAR 537
drSarm1 NYSTCDPNNLADWLADADPRFRQYTYGLVQSGVDRNNIVHITDQQLLTDCHVENGIHRAK 533

hsSARM1 ILTAAREMLHSPLPCTGGKPSGDTPDVFISYRRNSGSQLASLLKVHLQLHGFSVFIDVEK 597
drSarm1 ILSAARRPS---KPCLTD-SQPKGPDVFISYRRTTGSQLASLLKVHLQLRGFSVFIDVEK 589

hsSARM1 LEAGKFEDKLIQSVMGARNFVLVLSPGALDKCMQDHDCKDWVHKEIVTALSCGKNIVPII 657
drSarm1 LEAGRFEEKLITSVQRARNFILVLSANALDKCMGDVAMKDWVHKEIVTALNGKKNIVPVT 649

hsSARM1 DGFEWPEPQVLPEDMQAVLTFNGIKWSHEYQEATIEKIIRFLQGRSSRDSSAGSDTSLEG 717
drSarm1 DNFVWPDPTSLPEDMSTILKFNGIKWSHEYQEATIEKILRFLEGCPSQEKPDGAKTDKKE 709

hsSARM1 AAPMGPT      724
drSarm1 PQKK---      713
```

Blue: Residues predicted to be nitrated

Green: Cysteine residues with potential for nitrosylation

**Figure S2. SARM1 sequence alignment.** Alignment of human (hs) SARM1 and zebrafish (dr) Sarm1 protein sequences with tyrosine (Y) residues predicted to be nitrated (blue) and cysteine (C) residues predicted to be nitrosylated (green).

**a** Human SARM1 (PDB: 7LD0) ChainA

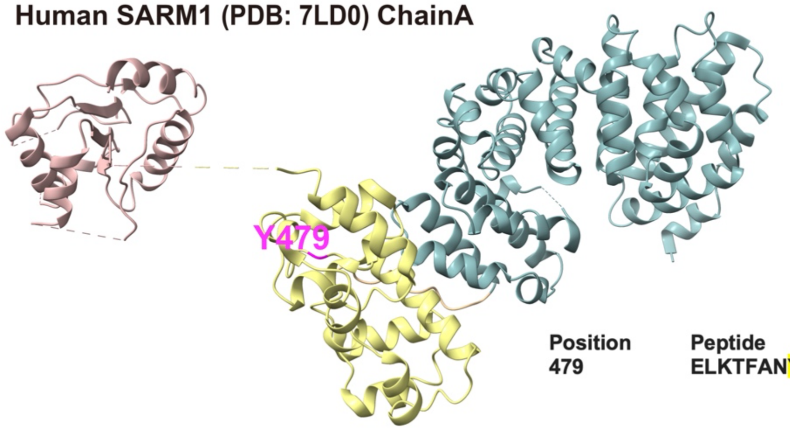


| Position | Peptide | Cluster |
|---|---|---|
| 479 | ELKTFANYSTCDRSN | Tyrosine Nitration |

**b** Zebrafish Sarm1(AlphaFold)

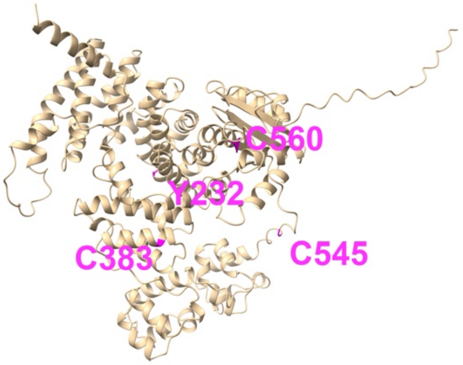


| Position | Peptide | Cluster |
|---|---|---|
| 232 | LSNCAMYGGHRCQR | Tyrosine Nitration |
| 383 | YSSNATVCSLAKRAL | S-Nitrosylation |
| 545 | ARRPSKPCLTDSQPK | S-Nitrosylation |
| 560 | GPDVFISYRRTTGSQ | Tyrosine Nitration |

**Figure S3. Prediction of nitration and nitrosylation in human and zebrafish SARM1**. (**a**) The crystal structure of human SARM1 Chain A (PDB 7LD0) shows that tyrosine (Y479) is predicted to be nitrated. The SAM domain is shown in yellow, the ARM domain is shown in teal, and the TIR domain is shown in pink. (**b**) Zebrafish Sarm1 modeling in AlphaFold predicts tyrosine residues (Y232 and Y560) to be nitrated and cysteine residues (Cys383, Cys545) to be nitrosylated.

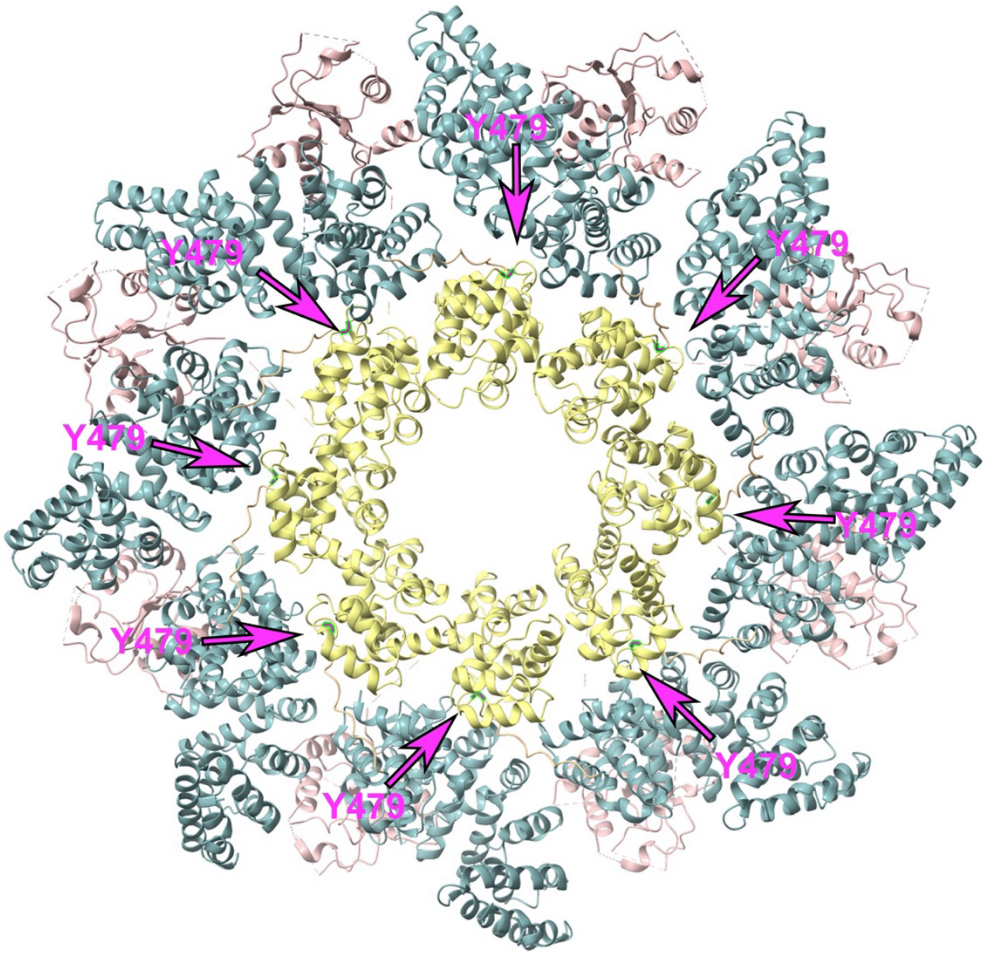


**Figure S4. Human SARM1 in octamer conformation**. The human SARM1 crystal structure (PDB 7LD0) is shown, and DeepNitro was used to analyze possible RNS modification. Tyrosine 479 is shown as a possible nitration site. The SAM domain is shown in yellow, the ARM domain is shown in teal, and the TIR domain is shown in pink. The Y479 is located at the SAM domain.

```
hsNMNAT1 SENSEKTEVVLLACGSFNPITNMHLRLFELAKDYMNGTGRYTVVKGIISPVGDAYKKKGL   60
drNmnat1 MASQEKIKLVLLACGSFNPITNMHLRMFELARDHLEDTGRYKVVKGIISPVGDGYKKKGL   60
hsNMNAT2 MTETTKTHVILLACGSFNPITKGHIQMFERARDYLHKTGRFIVIGGIVSPVHDSYGKQGL   60
drNmnat2 MTENTKTHVILLSCGSFNPITKGHIHMFEKAREYLHKTGRFIVIGGIVSPVHDSYGKPGL   60
hsNMNAT3 --MKSRIPVVLLACGSFNPITNMHLRMFEVARDHLHQTGMYQVIQGIISPVNDTYGKKDL   58
drNmnat3 --MAGRIPLVLLACGSFNPITHQHMRLFELARDHMHQTGLYRVVGGIISPVGDGYGKQGL   58

hsNMNAT1 IPAYHRVIMAELATKNSKWVEVDTWESLQKEWKETLKVLRHHQEKLEASDCDHQQNSPT-  119
drNmnat1 IE-CHRLEMARLATESSEWITVDDWESQQPEWVETAKVVRHHHAVLSSENSSNGDNVDT-  118
hsNMNAT2 VSSRHRLIMCQLAVQNSDWIRVDPWECYQDTWQTTCSVLEHHRDLMKRVTGCILSNVNTP  120
drNmnat2 VPSRHRLTMCQLAVQSSDWIRVDPWECYQDTWQTTCSVLEHHRDLMKRVTGCILSNVNTP  120
hsNMNAT3 AASHHRVAMARLALQTSDWIRVDPWESEQAQWMETVKVLRHHHSKLLRSPPQMEG-----  113
drNmnat3 VASKHRLAMARLALQSSDWVSVDDWESQQPDWTETVVTMRYHYGRVAAQHCCNKG-----  113

hsNMNAT1 -----------------------LERPGRKRKWTETQDSSQKKSL-EPK-----------  144
drNmnat1 -----------------------G----KYRKRRKM-EKKSPSCM-NPK-----------  138
hsNMNAT2 SMTPVIGQPQNETPQPIYQNSNVATKPTAAKILGKVGESLSRICCVRPPVERFTFVDENA  180
drNmnat2 STTPVIGQPQNETSA-IYQN--TVNKSVAIKFWGKMSESLGKICCVRPHMDRFTFVDENA  177
hsNMNAT3 --------------------------------------PDHGKAL-FST-----------  123
drNmnat3 --------------------------------------P--------PT-----------  116

hsNMNAT1 --------TKAVPKVKLLCGADLLESFAVPNLWKSEDITQIVANYGLICVTRAGNDAQKF  196
drNmnat1 ---------ADHSHLNLLCGADVLESFGVPNLWKPEDIEEIVGRYGVTCITRCGSDAEKF  189
hsNMNAT2 NLGTVMRYEEIELRILLLCGSDLLESFCIPGLWNEADMEVIVGDFGIVVVPRDAADTDRI  240
drNmnat2 NLGTAMRYEEIELRILLLCGSDLLESFCIPGLWNESDMEVIVGDFGIVVVPRDGADTERI  237
hsNMNAT3 --------PAAVPELKLLCGADVLKTFQTPNLWKDAHIQEIVEKFGLVCVGRVSHDPKGY  175
drNmnat3 --------TSDVPQLKLLCGADFMDSFKVPGLWTDEHIEEVVGRFGLVCVSRGSLQPDRA  168

hsNMNAT1 IYESDVLWKHRSNIHVVNEWIANDI---SSTKIRRALRR--GQSIRYLVPDLVQEYIEKH  251
drNmnat1 INQSDVLYKHRKNISVVREWVTNEI---SATHIRRALRR--GQSVRYLLPDSVVRYIQDH  244
hsNMNAT2 MNHSSILRKYKNNIMVVKDDINHPMSVVSSTKSRLALQHGDGHVVDYL-SQPVIDYILKS  299
drNmnat2 MNHSSVLRKHKDNIIVVKDEIDHPMSIVSSTKSRLALQHGDGHVVDYL-SQPVIDYILQS  296
hsNMNAT3 IAESPILRMHQHNIHLAKEPVQNEI---SATYIRRALGQ--GQSVKYLIPDAVITYIKDH  230
drNmnat3 IHESDLLSKHRPSIFLVREWVHNEI---SATEIRRALRR--GHSVKYLLPDSVIEYIREH  223

hsNMNAT1 NLYSSESEDRNAGVILAPLQRNTAEAKT       279
drNmnat1 SLYSAESEQKNAGVILAPLQRYTNTE--       270
hsNMNAT2 QLYINASG--------------------       307
drNmnat2 QLYINASG--------------------       304
hsNMNAT3 GLYTKGSTWKGKSTQSTEGKTS------       252
drNmnat3 KLYTQDSEMKNKDVKLRPLTKQVILD--       249
```

Yellow: Oxidation-prone cysteine residues with potential to form disulfide bonds

Blue: Residues predicted to be nitrated

Gray: Cysteine residues with potential for disulfide bond formation or nitrosylation

**Figure S5. Sequence alignment of human and zebrafish NMNAT proteins.** Yellow highlights cysteine residues with the potential to form disulfide bonds. Blue highlights residues that are predicted to be nitrated. Gray highlights cysteine residues with the potential of either disulfide bond formation or nitrosylation.

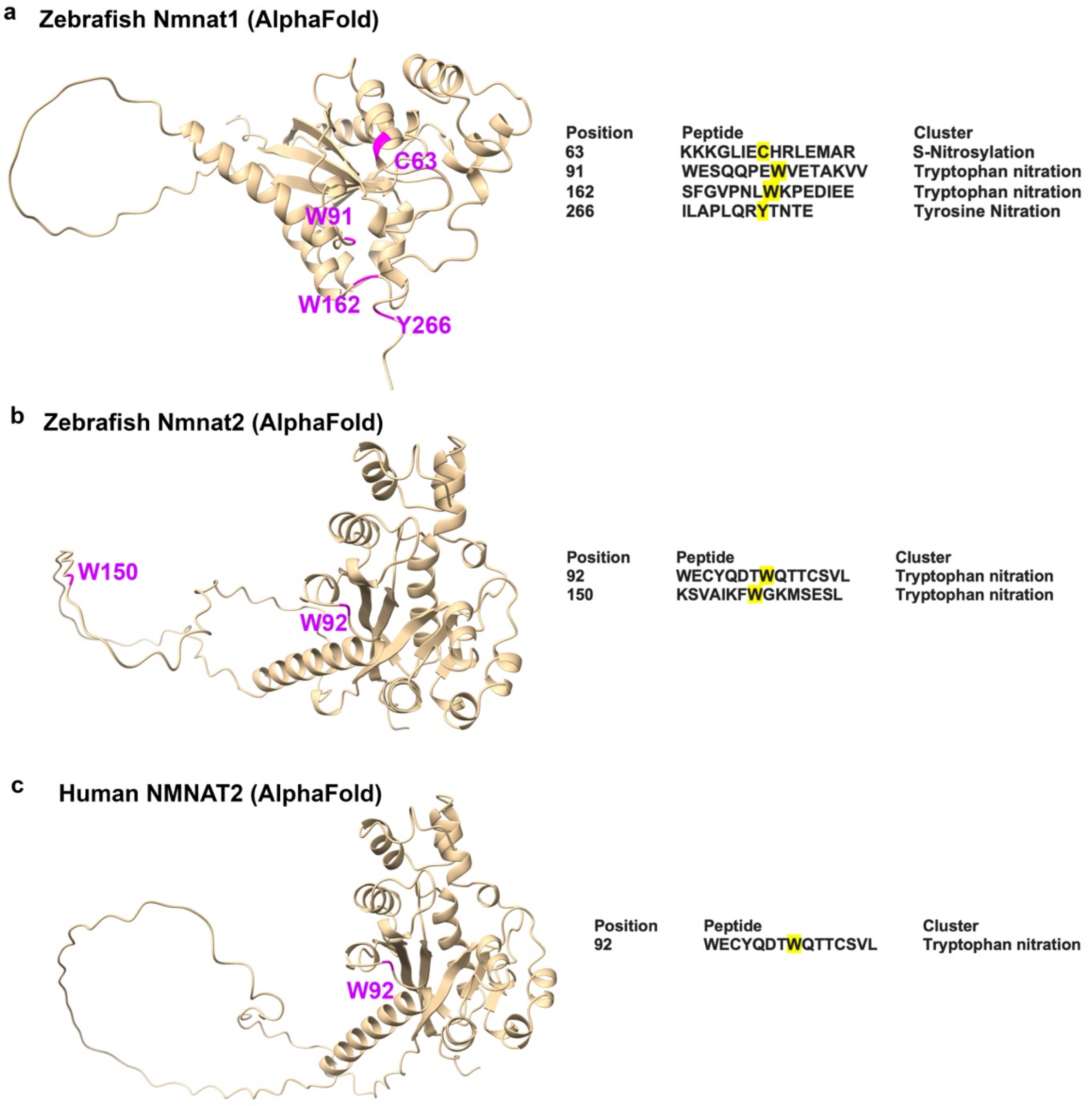


**Figure S6. Prediction of nitration and nitrosylation in human NMNAT2 and zebrafish Nmnat1/2**. (**a**) Zebrafish Nmnat1 was modeled in AlphaFold followed by DeepNitro analysis to predict residues that undergo nitration (Y or W) or nitrosylation (Cys). Predictions show W92/162, Y266, and Cys63 as possible sites for RNS modifications. (**b**) Zebrafish Nmnat2 modeled in AlphaFold and analyzed in DeepNitro shows possible nitration at W92 and W150. (**c**) Human NMNAT2 modeled in AlphaFold and analyzed with DeepNitro shows W92 as potentially nitrated residue.

**Likelihood of disulfide bond formation (<3Å)**

| C position | C113 | C229 | C346 | C478 | C523 | C621 |
|---|---|---|---|---|---|---|
| **C113** | | 23.289 | 30.114 | 58.735 | 60.801 | 52.087 |
| **C229** | | | 27.413 | 43.046 | 43.450 | 41.198 |
| **C346** | | | | 34.932 | 42.475 | 39.409 |
| **C478** | | | | | 13.527 | 51.967 |
| **C523** | | | | | | 60.227 |
| **C621** | | | | | | |

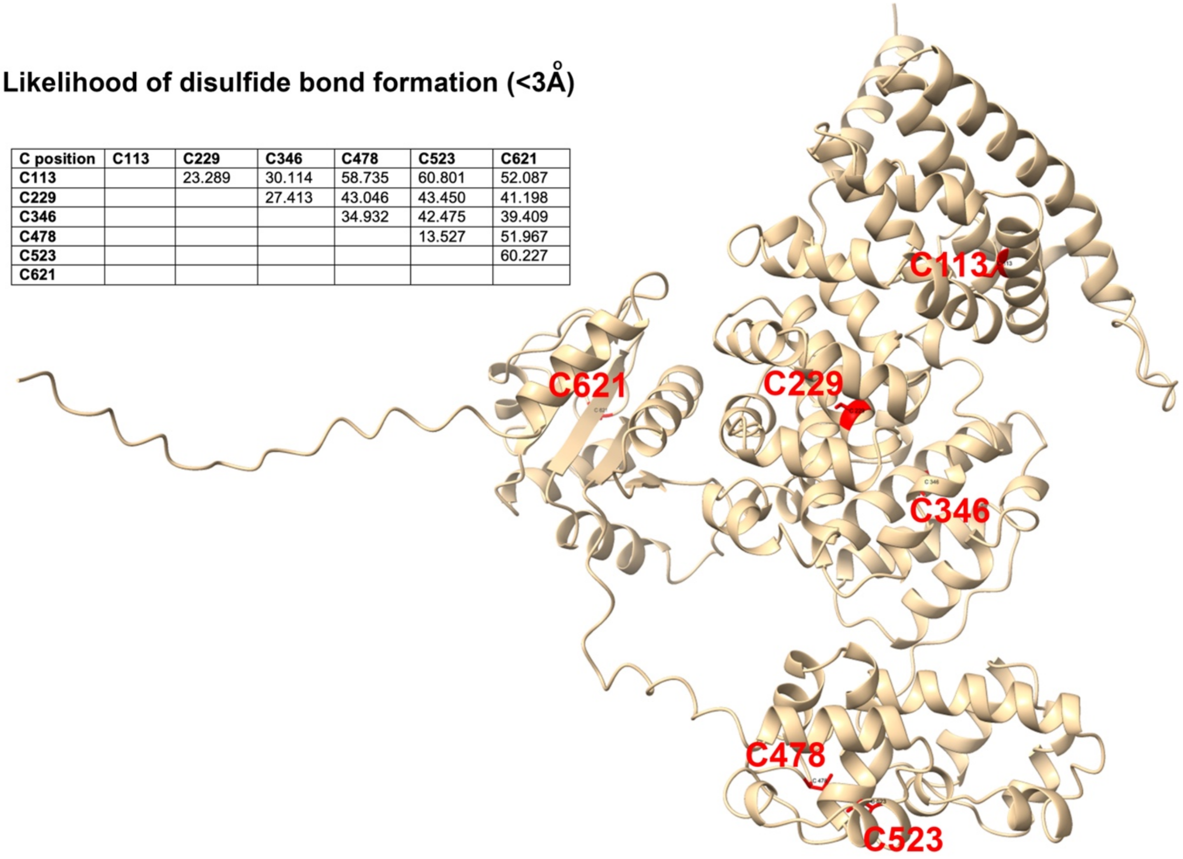


**Figure S7. Modeling disulfide bonds in zebrafish Sarm1 shows that disulfide bond formation between conserved cysteines is unlikely.** The zebrafish Sarm1 protein structure was generated in AlphaFold and analyzed with ChimeraX for possible cysteine oxidation sites that can participate in disulfide bond formation. Conserved cysteines 113, 229, 346, 478, 523, and 621 are shown in red. The distances between those cysteine residues are shown in the table and are indicated in Ångstrom.

| C position | C14 | C63 | C134 | C148 | C178 | C182 |
|---|---|---|---|---|---|---|
| C14 | | 19.477 | 36.636 | 9.277 | 13.896 | 25.240 |
| C63 | | | 51.087 | 26.272 | 29.919 | 34.566 |
| C134 | | | | 39.198 | 41.279 | 54.830 |
| C148 | | | | | 4.686 | 17.610 |
| C178 | | | | | | 14.166 |
| C182 | | | | | | |

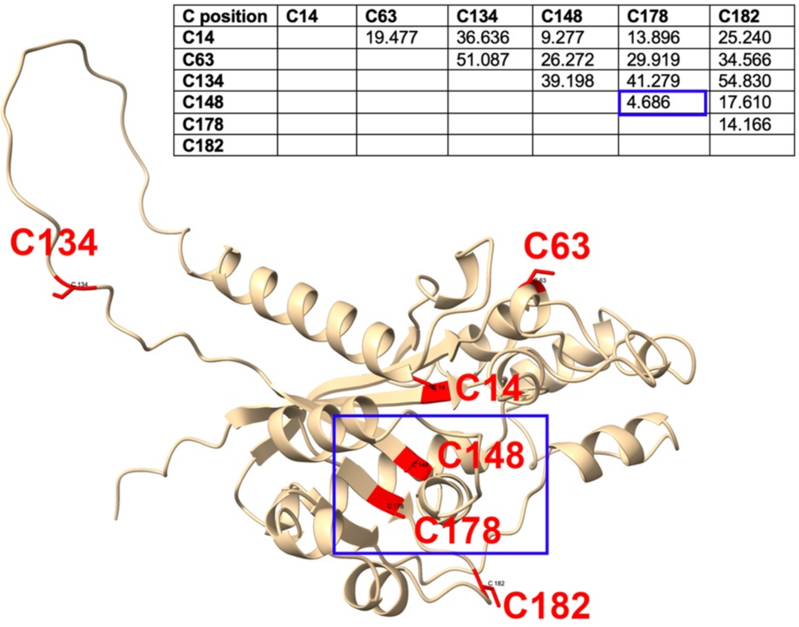


**Figure S8. Disulfide bond prediction shows possible bond formation by oxidation in zebrafish Nmnat1.** Zebrafish Nmnat1 generated via AlphaFold was analyzed in ChimeraX for conserved cysteine residues 14, 63, 134, 178, and 182 (shown in red). The distance between these cysteine residues is shown in the table and indicated in Ångstrom. C178 and 148 could potentially form a disulfide bond when oxidized (4.7Å, blue box).

**a** **Human NMNAT2**

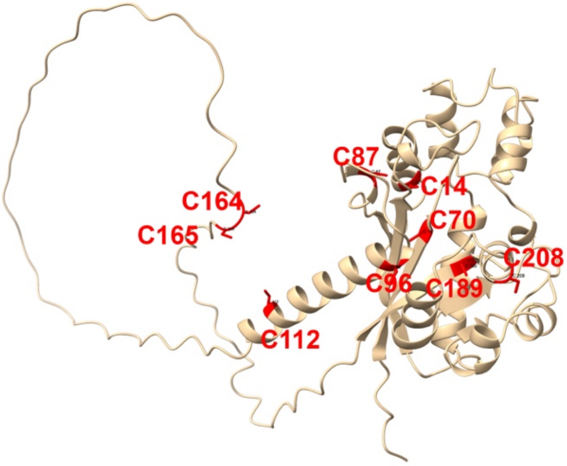


| C position | C14 | C70 | C87 | C96 | C122 | C164 | C165 | C199 | C208 |
|---|---|---|---|---|---|---|---|---|---|
| C14 | | 11.521 | 10.377 | 10.314 | 23.537 | 23.751 | 27.018 | 9.760 | 18.505 |
| C70 | | | 8.026 | 21.683 | 27.922 | 24.176 | 29.243 | 16.478 | 26.155 |
| C87 | | | | 18.256 | 25.511 | 20.131 | 25.307 | 18.689 | 26.155 |
| C96 | | | | | 23.662 | 26.581 | 27.969 | 14.250 | 17.450 |
| C112 | | | | | | 12.895 | 9.598 | 30.516 | 39.143 |
| C164 | | | | | | | 6.394 | 32.890 | 41.744 |
| C165 | | | | | | | | 35.674 | 44.157 |
| C199 | | | | | | | | | 11.582 |
| C208 | | | | | | | | | |

**b** **Zebrafish Nmnat2**

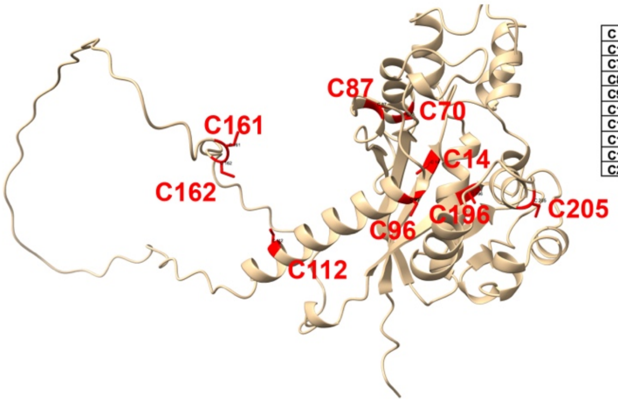


| C position | C14 | C70 | C87 | C96 | C112 | C161 | C162 | C196 | C205 |
|---|---|---|---|---|---|---|---|---|---|
| C14 | | 11.540 | 10.379 | 10.335 | 23.325 | 26.737 | 26.570 | 9.706 | 18.495 |
| C70 | | | 8.007 | 21.706 | 27.646 | 28.879 | 29.583 | 16.439 | 26.116 |
| C87 | | | | 18.230 | 25.283 | 22.905 | 24.915 | 18.610 | 26.522 |
| C96 | | | | | 23.567 | 27.080 | 26.612 | 14.309 | 17.554 |
| C112 | | | | | | 17.073 | 10.729 | 30.322 | 39.062 |
| C161 | | | | | | | 7.160 | 36.237 | 43.394 |
| C162 | | | | | | | | 35.411 | 43.309 |
| C196 | | | | | | | | | 11.596 |
| C205 | | | | | | | | | |

**Figure S9. Modeling disulfide bonds in human and zebrafish NMNAT2.** (**a**) Human NMNAT2 generated in AlphaFold and analyzed with ChimeraX shows conserved cysteine residues 14, 70, 87, 96, 122, 164, 165, 199, and 208 (shown in red). The distances between those cysteine residues are shown in the table in Ångstrom. (**b**) Zebrafish Nmnat2 generated in AlphaFold and analyzed with ChimeraX shows conserved cysteine residues 14, 70, 87, 96, 112, 161, 162, 196, and 205 (shown in red). The distances between those cysteine residues are shown in the table in Ångstrom.
